# Epidemiology and Clinical Burden of Meningococcal Disease in France: Scoping Review

**DOI:** 10.3390/jcm12030849

**Published:** 2023-01-20

**Authors:** Alexiane Baloche, Claude Dussart, Pierrick Bedouch, Florence Carrouel, Gérard Mick

**Affiliations:** 1 Health Systemic Process (P2S), Unit Research UR4129, University Claude Bernard Lyon 1, University of Lyon, 69008 Lyon, France; 2 University Grenoble Alpes, CNRS, UMR 5525, VetAgro Sup, Grenoble INP, TIMC Research Laboratory, 38700 Grenoble, France; 3 Hospices Civils de Lyon, 69002 Lyon, France; 4 Pharmacy Departement, CHU Grenoble Alpes, 38700 Grenoble, France; 5 Pain Center, Voiron Hospital, CHU Grenoble Alpes, 38500 Grenoble, France

**Keywords:** meningococcal diseases, epidemiology, mortality, sequelae, France

## Abstract

Invasive meningococcal disease (IMD) remains a significant health concern due to its unpredictable nature and its rapid progression. Even if occurrence of IMD is strictly monitored by a national surveillance network, no information on long-term sequelae is reported, making it difficult to assess the entire clinical burden of IMD in France. The aim of this scoping review was to analyze the epidemiology and the clinical burden of IMD in France by reporting the main epidemiological parameters, and by describing the clinical consequences and the care pathway of patients. The process of the review followed the Preferred Reporting Items for Systematic Reviews and Meta-Analyses extension to the Scoping Reviews guidelines. In France, the incidence of IMD cases has been fluctuating over time, characterized by an overall downward trend linked to a decrease in Sg B cases and the introduction of mandatory vaccination against Sg C. Sg W cases increased in recent years (from 5% to 21% in 2019). The case fatality rate remained constant (6–12.9%). The most frequently reported sequelae were severe neurological disorder, epilepsy, and anxiety. However, data on sequelae and care pathways were scarce. Further research should concentrate on providing robust identification of sequelae and the subsequent impact on quality of life, as well as on the organization of optimal care and support for patients and their families.

## 1. Introduction

Invasive meningococcal disease (IMD) is caused by the gram-negative bacterium *Neisseria meningitidis (N. meningitidis)* [1]. Twelve serogroups (Sg) of *N. meningitidis* have been identified with six Sg (A, B, C, W, X and Y) being responsible for most IMD cases worldwide [2]. The clinical presentation of IMD is usually diverse, with meningitis and septicemia being the most common presentations [3].

In Europe, IMD is a notifiable disease under surveillance [4]. The overall incidence in the general population in 2019 was 0.57/100,000. The main burden was highest in infants and young children, with a notification rate of 7.2/100,000 in children under one year of age, and 2.0/100,000 in 1–4-year-olds [4]. Sg B remains the most common cause of IMD in European countries [2], whereas, since the introduction of routine vaccination for Sg C, a decline in the notification rate of Sg C was observed [5]. Some European countries reported an increase of cases due to Sg Y and Sg W [5].

IMD remains a significant health concern due to its unpredictable nature and its rapid progression [6]. Missing the diagnosis is still feared by all practitioners in the early stage of the infection when the symptoms are nonspecific and indistinguishable from any other general infection syndromes [7]. Even when timely diagnosis and treatment are provided, the case fatality rate still ranges from 8% to 20% [8]. IMD survivors can suffer from reversible or irreversible outcomes in up to 20% of cases [7]. The range of sequelae is wide, including psychological and behavioral sequelae in addition to physical and neurological sequelae [7,9]. Even without identified sequelae, IMD leads to reduced quality of life in survivors, their families, caregivers, and the surrounding network [10].

To reduce this burden, the World Health Organization (WHO) set out a global roadmap in 2020 defining five pillars for IMD: (1) prevention and epidemic control; (2) diagnosis and treatment; (3) surveillance; (4) support and care; and (5) advocacy and engagement [11]. The fourth pillar particularly focuses on strengthening the support and care for families and survivors. The roadmap also proposes to conduct research on the socioeconomic impact of sequelae on children, adults, their families/careers, and on the availability and effectiveness of aftercare or support interventions.

In France, IMD is also a notifiable infection disease due to the potential epidemic spread [12]. IMD is strictly monitored by *Santé Publique France* (SPF) and *National Reference Center for Meningococci and Haemophilus influenzae* (NRCMHi) [12]. However, the French surveillance system reports no information on long-term sequelae, making it difficult to assess the entire burden of IMD in France. Moreover, considering the fourth pillar of the WHO roadmap, it will be useful to describe hospitalizations details and follow-up modalities of survivors.

This study aims to analyze the epidemiology and clinical burden of IMD in France by reporting the main epidemiological parameters of meningococcal infection and describing the clinical consequences of IMD and the care pathways of patients.

## 2. Materials and Methods

### 2.1. Design

The study was designed as a scoping review to provide an overview of the available research evidence [13,14]. This scoping review was performed following the Preferred Reporting Items for Systematic reviews and Meta-Analyses extension to the Scoping Reviews (PRISMA-ScR) methodological guidelines [15]. This methodology [16] allows inclusion of all study design if the following steps are respected: (1) identification of a research objective and search strategies; (2) selection of relevant publications; (3) categorization of the publications; and (4) summarizing, analyzing, and reporting results.

### 2.2. Research Question

To better understand the substantial IMD burden in France, this review was guided by the following questions: (1) What is the epidemiology of IMD? (2) What is the clinical burden of IMD, including sequelae? (3) What are the care pathways of patients with IMD?

### 2.3. Searching Strategy

The research was conducted from 20 May 2022 to 6 June 2022 in three databases (PubMed, Web of Science and Embase). The search terms were: “*Neisseria meningitidis*” OR “meningococcal infections” AND “epidemiology” OR “morbidity” OR “mortality OR “incidence” OR “prevalence” OR “hospitalization” AND “France”. More details are described in Tables S1–S3. After merging the results of the three databases, the duplicates were eliminated.

The eligibility of articles was checked by analyzing titles and abstracts. Articles were included if they were: (1) publications published from 2000 to 2022; (2) publications written in English or in French; (3) fully accessible without restriction; and (4) composed data on the epidemiology and clinical outcomes in France. The articles were excluded if they were: (1) posters, commentaries, letters, editorials, and (2) with no additional substantial value.

The screening and selection of publications was done independently by two investigators (AB and FC) based on predefined inclusion and exclusion criteria. After cross-checking of selected articles, discrepancies were solved by including a third investigator (GM).

### 2.4. Categorization Strategy

The publications included in the review were classified using the GRADE process [17], applied to evaluate the quality of the studies. The two reviewers independently graded the publications using the following levels: (1) High: the real effect is similar to that of the estimated effect; (2) Moderate: the real effect is likely to be similar to the estimated effect, but it may be considerably different; (3) Low: the real effect may be considerably different from the estimated effect; (4) Very low: the real effect is likely to be considerably different from the estimated effect.

### 2.5. Data Extraction

Data extraction was performed by one investigator (AB) and verified by a second (FC). The results were summarized by study design, study population, study date, type of endpoint, endpoint definition, and results or main findings.

## 3. Results

### 3.1. Study Selection Process

**Figure 1:**
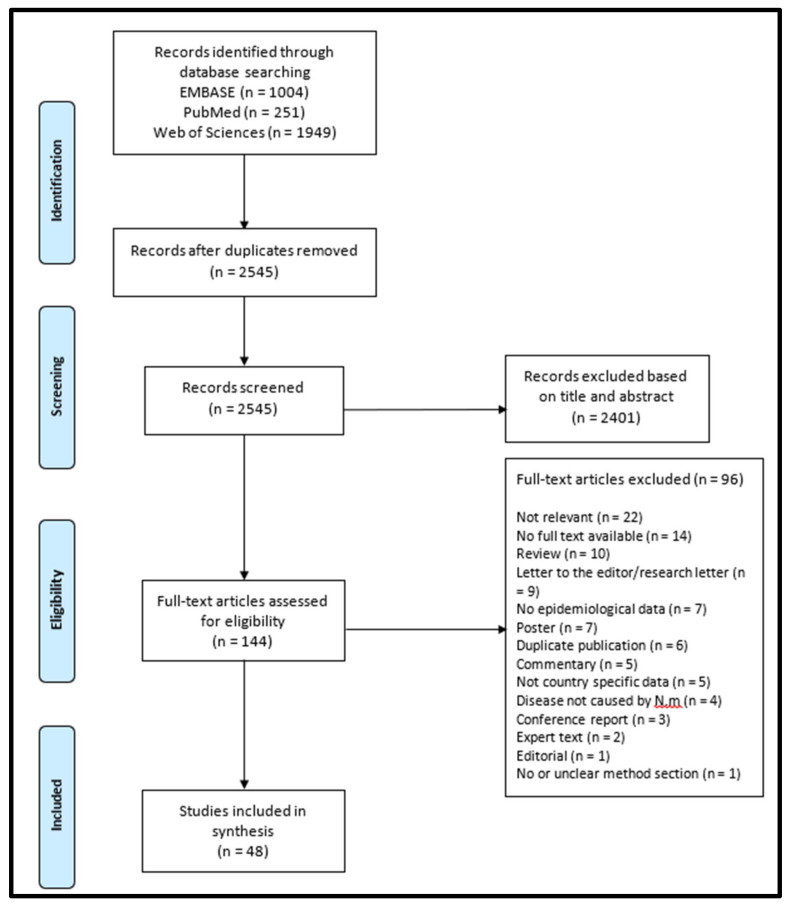
Flowchart of study and selection process.

The PRISMA-ScR study flowchart is presented in Figure 1. From initial database research, 3204 papers were identified. After duplicates were removed, 2545 papers were screened at title and abstract level, and 144 potentially relevant full text articles were selected for assessment of their eligibility. Finally, 48 publications fulfilled the inclusion criteria and were included in the review.

### 3.2. Characteristics of Publications Included

#### 3.2.1. Data sources and Scope of Included Studies

Most of studies (n = 30) were based on mandatory national surveillance data which is monitored by Santé Publique France and supported by the National Reference Center for Meningococci and *Haemophilus influenzae* (NRCMHi). Eleven articles used data from the French active bacterial pediatric meningitis surveillance network (*Groupe de Pathologie Infectieuse Pédiatrique*, GPIP/*Association Clinique et Thérapeutique Infantile du Val de Marne*, ACTIV) which gives clinical and biological features of bacterial meningitis in children. Five studies used the national health insurance data base (SNDS). Some studies included other data sources (medical records, interviews, or questionnaires).

Most publications reported national data (n = 29). One publication reported both national and regional data. Publications covering regional data mainly described data from northern regions of France (n = 10). Paris was the region most often cited (n = 6).

#### 3.2.2. Outcomes of Included Studies

The studies included had different outcomes. The most reported were epidemiological (n = 44), clinical (n = 37), case fatality rate (CFR) (n = 33), and IMD incidence and/or the number of notified IMD cases (n = 30), whereas clinical presentation (n = 21) and long-term sequelae (n = 13) were less frequently reported upon. Table S4 provides an overview of the included studies and their results.

#### 3.2.3. Quality of Included Studies

**Figure 2:**
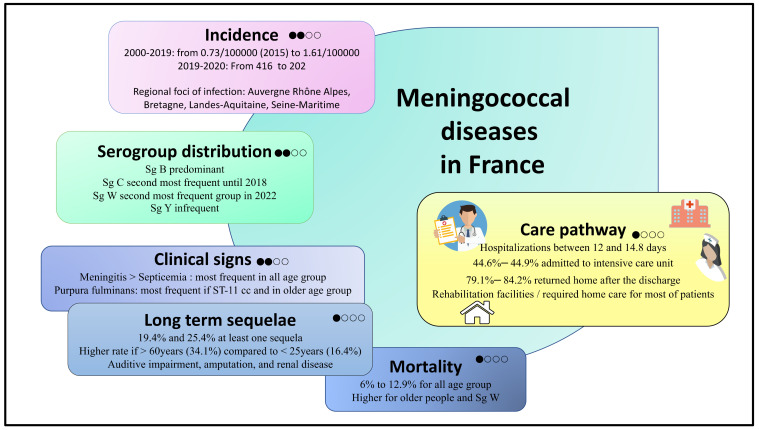
Summary of the results based on the GRADE process. High, when the true effect lies close to that of the estimate of the effect; moderate, when the true effect is likely to be close to the estimated effect, but there is a possibility that is substantially different; low, when true effect may be substantially different from the estimated effect; and very low, when the true effect is likely to be substantially different from the estimated effect. Sg: Serogroup; cc: clonal complex.

Figure 2 provides a summary of the results. No evaluated outcomes had a high level of GRADE ranking. Incidence, serogroup distribution, clinical presentation and CFR had low levels of evidence. Long-term sequelae, hospitalization details and long-term follow-up had very low levels of evidence.

### 3.3. Results of Included Studies

#### 3.3.1. Epidemiology of IMD in France

In France, an IMD case is defined as the presence of at least one of the following notification criteria: (1) isolation of *N. meningitidis* or a positive PCR test in blood, cerebrospinal fluid (CSF), or other sterile anatomical sites (e.g., joint, pleural, peritoneal, pericardial or aqueous fluid) or purpuric skin lesions; (2) detection of Gram-negative stained diplococcus in CSF; (3) purulent CSF associated with purpuric skin lesions; and (4) purpura fulminans (severe sepsis with extensive hemorrhagic and at least necrotic skin lesions of more than 3 mm diameter) [12].

IMD incidence rate defined as the total number of IMD cases per 100,000 individuals exposed to the risk of IMD was reported in 17 publications, all of them based on mandatory national surveillance data. According to the results, the incidence of IMD has been cyclical and dynamic in nature and has varied over time (Table S5). From 2000 to 2019, the overall incidence rate ranged between 0.73/100,000 and 1.61/100,000 by year [18,19]. In 2020, the significant decrease (from 416 in 2019 to 202 in 2020) seemed to be related to strict social restrictions implemented in France related to the *SARS-COV2* epidemic, including national lockdowns, universal outdoor masking, and social distancing [20]. Regardless of the pandemic situation, the principal age groups affected were infants (<1 year old), young children (1–4 years old), adolescents and young adults (15–24 years old), as well as the elderly (>65 years old) [20]. The overall incidence also varied geographically (Table S6). It was mainly related to several outbreaks that occurred in specific regions, such as Auvergne Rhône Alpes [21], Brittany [22], Landes-Aquitaine [23], and Seine-Maritime [24].

Most included studies specified the distribution by Sg (n = 29) or reported data on a specific Sg (n = 20). Sg B (n = 32), followed by Sg C (n = 28) and Sg W (n = 24) were the most examined Sg.

Sg B was predominant in France. The incidence of Sg B fluctuated between 2000 and 2007, characterized by a sudden increase between 2003 and 2005 linked to a localized hyperendemia in Seine-Maritime [25]. Since 2008, annual incidence rates of Sg B have decreased (0.69 cases per 10,000 inhabitants in 2006 to 0.39 in 2015) [19].

Sg C was the second most frequent serogroup until 2018. The incidence increased until 2002 (with an incidence rate of 0.5 cases per 100,000 inhabitants and a proportion of Sg C isolates of 41%) and then decreased [18]. Isolates belonging to the clonal complex 11 (cc11), considered to be highly invasive and lethal, have emerged since 2000 and were mainly associated to Sg C [18,26]. The emergence of this new strain raised concern in 2008 [18] about the probable arrival of a new epidemic cycle, which in fact started in 2011 [19]. Thus, routine vaccination against Sg C was recommended firstly in 2010, with one dose at 12 months, and secondly in 2017, with one dose at 5 months and one dose at 12 months as well as a catch-up until 24 years of age (one dose of Men C vaccine). Since 2018, a decrease in the number of Sg C cases was observed in infants, attributed to vaccination mandates (17 cases during 2012–2017 to 4 cases in 2018) [27]. The proportion of Sg C isolates also decreased to 24.3% in 2018, and to 14.8% in 2019, in all age group [28].

Sg W showed variations over the study period. An initial increase of Sg W cases was globally observed in the beginning of the 2000s due to an outbreak associated with the Hajj (1% of all invasive strains in 1995 vs. 10.6% in 2000) [29]. Sg W decreased and was the least frequent group between 2006–2015 (4% of cases belonged to Sg W vs. 96% to Sg BCY) [19]. A changing pattern in the epidemiology of Sg W has been observed in 2015–2016 in relation to the spread of the “UK 2013-strain”, belonging to cc11 isolates [30]. Since 2016, the proportion of Sg W isolates increased from 4% to 26.3% in 2020 [28,30].

Sg Y remained infrequent, even if it increased from 3% between 1998 and 2008 to 15% between 2017–2021 [18,20]. This increase was due to a global increase in Europe, particularly marked in the Scandinavian countries [19]. Unlike other Sg, Sg Y did not immediately decrease after the emergence of COVID-19 [20]. Rosain et al. showed that the frequency of Sg Y was significantly higher in the population with terminal complement pathway deficiencies compared with the general population [31].

#### 3.3.2. The Clinical Burden of IMD in France

IMD may manifest in multiple clinical presentations. 21 publications reported the proportion of IMD cases by clinical presentation, 9 reported specific data for the pediatric population [21,32,33,34,35,36,37,38,39] and 2 for the adult population [40,41]. Meningitis followed by septicemia was the most globally common clinical presentation in all age groups (Table 1). Purpura fulminans were more frequent in the older age group (9.1–29.7% of cases in children vs. 36.9% of adult cases). Serogroup-specific distribution on the clinical presentation of IMD was based on data from mandatory notification. Authors reported that Sg W cases presented more frequently with septicemia and less frequently with meningitis in comparison with Sg B and Sg C cases in all age groups [30]. Two studies found higher rates of purpura fulminans with isolates belonging to the cc11 (42–73%) [18,42].

The CFR, defined as the total number of deaths from IMD on the total confirmed IMD cases, was reported in 33 publications, including 14 publications reporting national data (Table 2). It ranged between 6% and 12.9% overall for all age groups at a national level. Higher rates were reported for older people and Sg W. Weil-Olivier et al. found that the mortality risk after the discharge was highest for the 25–59 age group, compared with the controls without IMD (hazard ratio: 4.9 (95% CI: 3.1–7.7)) [46]. It also depended on the clinical presentation and the clonal complex. Contou et al. reported the highest CFR (36.9%) in adults diagnosed with purpura fulminans caused by *N. meningitidis* [47], whereas Floret reported the highest CFR (79%) in the pediatric population [35]. Sg W:cc11 was also associated with higher CFR (4.0–27.8%) [30].

The number of patients with long-term sequelae after IMD were reported in 13 publications. Three observational studies, of which two used health insurance data, reported the prevalence of certain sequelae (Table S7). The overall proportion of patients with at least one sequela for all age groups ranged between 19.4% and 25.4% (Table 3). The rates of sequelae depended on the follow-up time, with higher rates for long-term follow-up studies (i.e., 25.4% in 2.8 years follow-up study [46]). The rate of sequelae also depended on age and clinical presentation (Table 3). Weil-Olivier et al. found a higher rate in survivors aged >60 years (34.1%) compared with survivors aged <25 years (16,4%) [46]. Huang et al. reported that patients with both septicemia and meningitis most likely had at least one sequela (21.6%), followed by meningitis only (19.9%) and septicemia only (19.3%) [43].

IMD sequelae could be distributed into five categories: physical, neurological, cognitive, and psychological (Table 4). The most frequently reported sequelae were severe neurological disorder, epilepsy, and anxiety for all age groups. For certain sequela, the rate varied depending on clinical presentation or the study design. For instance, Weil-Olivier et al. reported 1.4–1.9% of adult cases (aged >19 years) with IMD having amputations [46]. Higher rates (11.6%) were reported in adult cases with *N. meningitidis* purpura fulminans [47]. In the same way, a prospective observational study found that 34.3% of adult cases had depressive symptoms one year after being discharged [40]. This outcome was not consistent with a case-control study reporting sequelae 2.8 years following IMD. In this study, 3.7% of adult cases had depressive symptoms [46]. Reduction of quality of life was mentioned in one observational study. Duval et al. reported that 50% and 30% of IMD patients had physical component or mental component scores lower than the 25th percentile of the score distribution in the French general population, respectively [40].

#### 3.3.3. Care pathways of Patients with IMD in France

During the acute phase, patients were usually admitted to hospital. Two studies used national health insurance data base (*Système National de Données de Santé*, SNDS) to describe the length of hospitalization, the proportion of Intensive Care Unit (ICU) admission and the discharge status after IMD [43,46]. They reported a median length of hospitalizations between 12 and 14.8 days. Around half of cases (44.6–44.9%) were admitted to ICU, and between 79.1% and 84.2% of cases returned home after the discharge.

After the acute phase and hospitalization, patients entered a phase with monitoring of possible sequelae. Three studies reported the follow-up modalities of patient surviving IMD [43,55,56]. Weil-Olivier et al. showed that most patients were admitted to rehabilitation facilities and required home care, comparing with controls without IMD. Additionally, in the year following hospitalization, they underlined that nearly two time more cases consulted a hospital-based specialist as an outpatient compared with controls without IMD. Cases also more frequently received nursing care, physiotherapy, and speech therapy compared with controls [56]. Huang et al. specified that patients with at least one sequela had more rehabilitation care than patients without sequelae (27.3% vs. 4.8%, respectively) [43].

## 4. Discussion

This scoping review aimed to provide an overview of the epidemiology and clinical burden of IMD in France. Most of the publications reported incidence data differentiated by Sg. In general, results on IMD incidence differed by the reporting year, age of subjects, and population investigated. However, data on sequelae were limited. Additionally, individual studies reported very different long-term sequelae rates depending on the design of the study, follow up time, and sequelae studied.

Concerning the epidemiology and clinical burden, this review shows that the incidence has been fluctuating over the period reviewed, but was still important in key age groups, such as infants. The gradual decreasing trend observed since 2008 was particularly linked to the decrease in Sg B cases [57]. The introduction of Men C vaccination in mandatory vaccinations in 2017 helped to reduce Sg C cases [57]. Notification data showed that, among the most frequent serogroups, cases with Sg Y and Sg W were the only types that have increased in recent years. Sg W accounted for <5% of all reported cases by 2014, but has grown steadily in recent years to 21% in 2019 [58]. The Sg Y portion also increased continuously during the observation period, but at a lower level. These observations were also reported in other European countries, such as the Netherlands [59], the UK [60], and Germany [61].

The CFR remained constant despite falling case numbers, with Sg W being a particularly important contributor to high mortality in all age groups [30]. In a recent article, the increase of Sg W:cc11 was found to be related to the increase of abdominal presentation of IMD. This nonspecific symptom contributes to the delay of management, and hence, a high rate of morbidity and mortality [62]. Compared with other European countries, the CFR was high (12% in 2019). For instance, in 2019, CFR was 8.1% in the UK, 9.2% in Germany, 10.3% in the Netherlands, and 11.3% in Spain [4].

The risk to survivors of developing severe and persistent sequelae was also significant. It was estimated that between 19.4% and 25.4% of survivors would have at least one sequela, highlighting that the burden of IMD went beyond the acute phase of the disease [43,46]. These findings are consistent with data reported in the literature for Germany (24% of survivors) [63] and Denmark (25% of survivors) [64]. The most frequent sequelae for all age group were neurological sequelae, such as epilepsy or severe neurological disorder, anxiety, and hearing impairment, which are also consistent with literature findings [9,10]. Nevertheless, the heterogeneity between studies in terms of design, study population, length of the follow-up, as well as definition and assessment tools used for sequelae, made difficult obtain a real estimation of sequelae proportions.

This review helps to highlight several gaps and suggested areas for future research. Firstly, regarding epidemiological data, the incidence and the mortality of IMD in France was well described in the literature. The mandatory national surveillance network, which published a yearly report on the epidemiological situation, strongly contributed awareness of the evolution of epidemiology [65]. Moreover, it is supported by other surveillance networks, such as the French active bacterial pediatric meningitis surveillance network (GPIP/ACTIV), which contributes to understanding the clinical features in the pediatric population [38]. However, there were few articles reporting specific data for the adult population. It will be interesting to have more information on this population, since the severity of the disease may be greater [46] and IMD prevalence seems to be shifting to older people [66].

Concerning the burden and severity of long-term sequelae associated with IMD, data are limited for France. Most publications reported the number of patients with at least one sequela or severe sequelae, only a few of them differentiated by types of sequelae. Additionally, most of these publications assessed physical and neurological sequelae, whereas data on psychological or behavior sequelae were very scarce. One reason which could explain this underreporting is the time needed for sequelae to develop. As it was demonstrated in a recent review, physical and neurological sequelae are likely to be reported because they are often more rapidly apparent than psychological or behavioral sequelae, which may be delayed in developing are poorly recognized [9]. As strong evidence of this, a recent case-control study reported the median time for psychological sequelae to develop is 15.5 months (vs 8.5 months for neurological sequelae and 1 month for physical sequelae), highlighting the necessity to have an extended and global follow-up of survivors [67]. Moreover, post-traumatic stress disorder or depression are likely easier to diagnose in adults than in young children. As early occurrence of such disorders can have a dramatic effect on childhood development [68], further investigations should focus on adequate ways for capturing sequelae proportion in all categories for all age groups.

Regarding patient-centered care, the question of the impact of IMD on survivors’ health-related quality of life (HRQL) arises. However, the available evidence is scarce. Only one observational study assessed the quality of life of adults survivors one year after the diagnosis, according to the research strategy of the present study [40]. Considering the patient’s subjectively perceived wellness may improve the speed and quality of recovery from IMD [69]. Data on the impact of IMD on caregivers are also very limited. As scientific literature describes the family burden of IMD [7,10], no observational study describes this aspect of the burden in France. Only one qualitative study assessed the impact of bacterial meningitis on caregivers [70], showing that bacterial meningitis involved changes in their professional trajectories and led to serious financial impacts on the family. Such an IMD impact obliges finding a new type of everyday functioning (adjustments concerning material aspects, such as person’s living and housing and the practical organization of childcare). This also concerns long-term psychological consequences linked to traumatic events (confrontation with the possibility of the child’s death, awareness of possible sequelae) [71]. Further research needs to be conducted on the global long-term burden of IMD in patients and their families to better understand the lifelong impact of the disease and to determine how health professionals can help them.

Finally, regarding the wide range of potential consequences of IMD, further research should concentrate on providing data on the organization of care and support for patients and their families after the acute phase in the long term. Just as failure to diagnose IMD in a time can have serious consequences, the non-systematic follow-up of IMD survivors can reduce the chance of a full recovery. An English case-control study reported that medical follow-up care of survivors was worryingly poor, and that, despite somatic deficits, only half of patients reported medical follow-up care by a health professional. Among them, 20% of those admitted to the ICU did not report receiving continuing medical care, and none of the 20% of case subjects with findings in the clinical range for depressive symptoms received a referral to an appropriate professional [71]. A better understanding of global disease management for survivors and their families may also help to identify the various aspects of care and support that could be optimized, with the aim of limiting the long-term burden of IMD.

The present study has some limitations. Due to the scope of this review being limited to French data, the transferability of the findings to other countries is limited. In addition, the high degree of heterogeneity regarding study designs, disease manifestations, ages of subjects, populations investigated, time points of data collection, and definition of sequelae makes difficult to compare findings. The representativeness of data is also questionable in terms of proportion of cases included in some studies (i.e., case study), the geographical coverage of some studies (i.e., regional study) as well as the risk of underreporting or potentially inaccurate reporting of diseases (mandatory surveillance data vs. health insurance data). Finally, although the search strategy of this review attempts to perform an accurate overview, it may not have located all available sources, especially those from grey literature sources that were not used in the data search.

## 5. Conclusions

This scoping review highlights the epidemiology and clinical burden of IMD in France, although data are limited, particularly regarding sequelae. Despite this, the IMD consequences appeared devastating to patients and their families. In the context of the WHO defined strategy for IMD, it is essential to seek recognition of IMD-induced disability, especially on a long-term basis and including all aspects of sequelae. Further research should concentrate on providing robust identification of sequelae, their impact on quality of life, as well as on the optimal organization of care and support for patients and their families.

## Figures and Tables

**Table 1:** Distribution of clinical presentation of IMD by age group in France.

	Proportion of IMD Diagnoses	References
Meningitis	43–79% (all ^1^) 61.1% (children ^2^) 56.5–63.1% (adult ^3^)	[19,43,44] [43] [40,43]
Septicemia	12.2–25.1% (all ^1^) 22.2% (children ^2^) 28.2% (adult ^3^)	[19,43,44] [43] [43]
Septicemia and meningitis	9.4–18% (all ^1^) 12.3% (children ^2^) 6.5% (adult ^3^)	[19,43,44] [43] [43]
Purpura fulminans	25.6–57% (all ^1^) 9.1–29.7% (children ^2^) 36.9% (adult)	[19,44] [37,38,45] [40]
Unspecified or other type of IMD	1.1–6.6% (all ^1^) 4.5% (children ^2^) 8.8% (adult ^3^)	[43,44] [43] [43]

^1^ All: both children and adult; ^2^ Children: patient aged <18 years old; ^3^ Adult: patient aged ≥18 years old.

**Table 2:** Case fatality rate (%) by serogroup, age group, clinical presentation, and hyperinvasive clonal complex.

	Case Fatality Rate ^1^	References
Overall	6.0–12.9% (all ^2^)	[19,43,48]
Serogroup		
B	7.8–8.8% (all ^2^) 5.3–8.2% (children ^3^)	[19,25,30] [39,49,50,51]
C	12.3–13.2% (all ^2^) 8.4–9.9% (children ^3^)	[19,30] [39,49,50,51]
W	11.9–22.1% (all ^2^) 6% (children ^3^)	[19,30,52] [34]
Y	15.5–17.2%	[19,30]
Age group		
<1 year	5.1–9.9% (all ^2^)	[19,37,38,46]
1–4 years	5.1–8.9% (all ^2^)	[19,38,46]
5–14 years	4.1–5.9% (all ^2^)	[19,46]
15–24 years	7.7–10.3% (all ^2^)	[19,46]
25–59 years	9.3% (all ^2^)	[19]
>60 years	20% (all ^2^)	[19]
Clinical presentation		
Meningitis only	5.6% (all ^2^) 21% (children ^3^)	[43] [35]
Septicemia only	7.7% (all ^2^)	[43]
Both septicemia and meningitis	3.9% (all ^2^)	[43]
Purpura fulminans	36.6% (all ^4^) 36.9% (adults ^4^) 79% (children ^3^)	[44] [47] [35]
Hyperinvasive clonal complex 11		
Overall	16% (all ^2^)	[26]
Sg C	22% (all ^2^)	[18]
Sg W	4.0–27.8% (all ^2^)	[30]

^1^ Total number of deaths from IMD on the total confirmed IMD cases; ^2^ All: both children and adults; ^3^ Children: patient aged <18 years old; ^4^ Adult: patient aged ≥18 years old.

**Table 3:** Proportion of patients with at least one sequela associated with invasive meningococcal disease by age group.

		Rate	References
Age group	All ages	19.4–25.4%	[43,46,53]
	Children (<18 years)	9.3–15.3%	[46]
	Adult (≥18 years)	11.3–31.3%	[40,46]
Clinical presentation	Meningitis	19.9%	[43]
	Septicemia	19.3%	[43]
	Both septicemia and meningitis	21.6%	[43]

**Table 4:** Type of sequelae associated with invasive meningococcal diseases.

Sequelae	Rate	References
**Physical**		
Amputations	1.5–2.7% (all ^1^) 1.4–11.6% (adults ^2^)	[43,46] [46,47]
Skin necrosis/scarring	2.3–2.7% (all ^1^)	[43,46]
Renal disease	1.3–1.9% (all ^1^)	[21,43,46]
Persistent gonaglias *		[54]
Mechanical arthralgias *		[54]
Persistent inflammatory syndrome *		[32]
**Sensorial**		
Hearing impairment	2.8–4.8% (all ^1^) 15.5% (adults ^2^)	[43,46] [40]
Severe visual impairment/blindness	1.7% (all ^1^)	[46]
**Neurological**		
Motor disorders	3.5% (all ^1^)	[46]
Epilepsy	5.8% (all ^1^)	[46]
Migraine/Headache	32.9% (adults ^2^)	[40]
Severe neurological disorder	5.5% (all ^1^)	[46]
Speech or communication problems	1.7% (all ^1^)	[46]
**Cognitive**		
Intellectual disability/ cognitive impairment	1.7% (all ^1^) 10% (adults ^2^)	[43] [40]
**Behavior or psychological**		
Sleep disorders	42.9% (adults ^2^)	[40]
Depressive symptoms	2.5% (all ^1^) 34.3% (adults ^2^)	[46] [40]
Anxiety	5.5% (all ^1^)	[46]

^1^ All: both children and adults; ^2^ Adult: patient aged ≥18 years old. * Sequelae reported in case study.

## Data Availability

No new data were created or analyzed in this study. Data sharing is not applicable to this article.

## Supplementary Materials

The following supporting information can be downloaded at: https://www.mdpi.com/article/10.3390/jcm12030849/s1. Table S1: Search strategy for PubMed; Table S2: Search strategy for EMBASE; Table S3: Search strategy for Web of Sciences; Table S4: Overview of study characteristics and results of included publications; Table S5: Characteristics of national studies including IMD incidence data distributed by age and serogroup; Table S6: Characteristics of regional studies including IMD incidence data distributed by age and serogroup; Table S7: Characteristics of included studies assessing and describing the type and the proportion of sequelae in France [72,73,74,75,76,77,78,79,80].
