# Supplementary material for: Epidemiology and Clinical Burden of Meningococcal Disease in France: Scoping Review
Source: J Clin Med. 2023 Jan 20;12(3):849. doi: 10.3390/jcm12030849 (PMC9917955; doi:10.3390/jcm12030849)
Supplement: Supplementary file 1 [file jcm-12-00849-s001.zip › jcm-2130297-supplementary.pdf]

Supplementary materials

Table S1. Search strategy for PubMED

#	Term	Search term	Number of results
1	“Meningococcal Infections” [MeSH]	"meningococcal infections"[MeSH Terms]	
2	“Meningitis, Meningococcal” [MeSH]	"meningitis, meningococcal"[MeSH Terms]	
3	“Neisseria Meningitidis” [MeSH]	"neisseria meningitidis"[MeSH Terms]	
4	“meningitis” [MeSH]	"meningitis"[MeSH Terms]	
5	#1 OR #2 OR #3 OR #4		67,910
6	Epidemiology [MeSH]	"epidemiology"[MeSH Terms]	
7	Morbidity [MeSH]	"morbidity"[MeSH Terms]	
8	Mortality [MeSH]	"mortality"[MeSH Terms]	
9	Incidence [MeSH]	"incidence"[MeSH Terms]	
10	Prevalence [MeSH]	"prevalence"[MeSH Terms]	
11	Hospitalization [MeSH]	"hospitalization"[MeSH Terms]	
12	#6 OR #7 OR #8 OR #9 OR #10 OR #11		1,262,117
13	#5 AND #12		5187
14	France	"france"[Title/Abstract]	
15	French	"french"[Title/Abstract]	
16	France (Affiliation)	"french"[Affiliation] OR "france"[Affiliation]	
17	#14 OR #15 OR #17		939,287
18	#13 AND #18		296
19	([english]/lim OR [french]/lim) AND [2000-2022]/py		
**20**	**#18 AND #19**		**251**

Table S2. Search strategy for EMBASE

#	Term	Search term	Number of results
1	Meningitis [Emtree]	'meningitis'/exp	
2	"Neisseria Meningitidis" [Emtree]	'neisseria meningitidis'/exp	
3	Meningococcemia [Emtree]	'meningococcemia'/exp	
4	meningitis	meningitis:ti,ab	
5	meningococcal	meningococcal:ti,ab	
6	meningococci	meningococci:ti,ab	
7	meningitidis	meningitidis:ti,ab	
8	meningitides	meningitides:ti,ab	
9	meningococcus	meningococcus:ti,ab	
10	meningococcemia	meningococcemia:ti,ab	
11	IMD	imd:ti,ab	
12	#1 OR #2 OR #3 OR #4 OR #5 OR #6 OR #7 OR #8 OR #9 OR #10 OR #11		162,511
13	Epidemiology [Emtree]	'epidemiology'/exp	
14	"Epidemiological Data" [Emtree]	'epidemiological data'/exp	
15	Morbidity [Emtree]	'morbidity'/exp	
16	Mortality [Emtree]	'mortality'/exp	
17	Incidence [Emtree]	'incidence'/exp	
18	Prevalence [Emtree]	'prevalence'/exp	
19	Hospitalization [Emtree]	'hospitalization'/exp	
20	epidemiology	epidemiology:ti,ab	
21	epidemiological	epidemiological:ti,ab	
22	epidemiologic	epidemiologic:ti,ab	
23	burden	burden:ti,ab	
24	incidence	incidence:ti,ab	
25	prevalence	prevalence:ti,ab	
26	hospitalization	hospitalization:ti,ab	
27	hospitalizations	hospitalizations:ti,ab	
28	hospitalisation	hospitalisation:ti,ab	
29	hospitalisations	hospitalisations:ti,ab	
30	complication	complication:ti,ab	
31	complications	complications:ti,ab	
32	sequelae	sequelae:ti,ab	
33	mortality	mortality:ti,ab	
34	morbidity	morbidity:ti,ab	
35	"natural history"	'natural history':ti,ab	
36	"course of disease"	'course of disease':ti,ab	
37	"disease course"	'disease course':ti,ab	
38	surveillance	surveillance:ti,ab	
39	outbreak	outbreak:ti,ab	
40	outbreaks	outbreaks:ti,ab	
41	death	death:ti,ab	
42	deaths	deaths:ti,ab	
43	died	died:ti,ab	
44	survival	survival:ti,ab	
45	fatality	fatality:ti,ab	
46	fatal	fatal:ti,ab	
47	#13 OR #14 OR #15 OR #16 OR #17 OR #18 OR #19 OR #20 OR #21 OR #22 OR #23 OR #24 OR #25 OR #26 OR #27 OR #28 OR #29 OR #30 OR #31 OR #32 OR #33 OR #34 OR #35 OR #36 OR #37 OR #38 OR #39 OR #40 OR #41 OR #42 OR #43 OR #44 OR #45 OR #46		9,213,111
48	#12 AND #47		69,387
49	France [Emtree]	'france'/exp	
50	French (citizen) [Emtree]	'french (citizen)'/exp	
51	france	france:ti,ab,ff	
52	French	french:ti,ab,ff	
53	#49 OR #50 OR #51 OR #52		371,674
54	#48 and #53		1234
55	([english]/lim OR [french]/lim) AND [2000-2022]/py		
**56**	**#54 AND #55**		**1004**

Table S3. Search strategy for Web of Sciences

#	Term	Search term	Number of results
1	Meningitis	ALL=(meningitis)	
2	"Neisseria Meningitidis"	ALL=("neisseria meningitidis)	
3	Meningococcemia	ALL=(meningococcemia)	
4	meningitis	(TI=(meningitis)) OR AB=(meningitis))	
5	meningococcal	(TI=(meningococcal)) OR AB=(meningococcal))	
6	meningococci	(TI=(meningococci)) OR AB=(meningococci))	
7	meningitidis	(TI=(meningitidis)) OR AB=(meningitidis))	
8	meningitides	(TI=(meningitides)) OR AB=(meningitides))	
9	meningococcus	(TI=(meningococcus)) OR AB=(meningococcus))	
10	meningococcemia	(TI=(meningococcemia)) OR AB=(meningococcemia))	
11	IMD	(TI=(IMD)) OR AB=(IMD))	
12	#1 OR #2 OR #3 OR #4 OR #5 OR #6 OR #7 OR #8 OR #9 OR #10 OR #11		71,759
13	Epidemiology	ALL=(epidemiology)	
14	"Epidemiological Data"	ALL=("epidemiological data")	
15	Morbidity	ALL=("morbidity")	
16	Mortality	ALL=("mortality")	
17	Incidence	ALL=("incidence")	
18	Prevalence	ALL=("prevalence")	
19	Hospitalization	ALL=("hospitalization")	
20	epidemiology	(TI=(epidemiology)) OR AB=(epidemiology))	
21	epidemiological	(TI=(epidemiological)) OR AB=(epidemiological))	
22	epidemiologic	(TI=(epidemiologic)) OR AB=(epidemiologic))	
23	burden	(TI=(burden)) OR AB=(burden))	
24	incidence	(TI=(incidence)) OR AB=(incidence))	
25	prevalence	(TI=(prevalence)) OR AB=(prevalence))	
26	hospitalization	(TI=(hospitalization)) OR AB=(hospitalization))	
27	hospitalizations	(TI=(hospitalizations)) OR AB=(hospitalizations))	
28	hospitalisation	(TI=(hospitalisation)) OR AB=(hospitalisation))	
29	hospitalisations	(TI=(hospitalisations)) OR AB=(hospitalisations))	
30	complication	(TI=(complication)) OR AB=(complication))	
31	complications	(TI=(complications)) OR AB=(complications))	
32	sequelae	(TI=(sequelae)) OR AB=(sequelae))	
33	mortality	(TI=(mortality)) OR AB=(mortality))	
34	morbidity	(TI=(morbidity)) OR AB=(morbidity))	
35	"natural history"	(TI=("natural history")) OR AB=("natural history"))	
36	"course of disease"	(TI=(" course of disease")) OR AB=(" course of disease"))	
37	"disease course"	(TI=(" disease course")) OR AB=(" disease course"))	
38	surveillance	(TI=(surveillance)) OR AB=(surveillance))	
39	outbreak	(TI=(outbreak)) OR AB=(outbreak))	
40	outbreaks	(TI=(outbreaks)) OR AB=(outbreaks))	
41	death	(TI=(death)) OR AB=(death))	
42	deaths	(TI=(deaths)) OR AB=(deaths))	
43	died	(TI=(died)) OR AB=(died))	
44	survival	(TI=(survival)) OR AB=(survival))	
45	fatality	(TI=(fatality)) OR AB=(fatality))	
46	fatal	(TI=(fatal)) OR AB=(fatal))	
47	#13 OR #14 OR #15 OR #16 OR #17 OR #18 OR #19 OR #20 OR #21 OR #22 OR #23 OR #24 OR #25 OR #26 OR #27 OR #28 OR #29 OR #30 OR #31 OR #32 OR #33 OR #34 OR #35 OR #36 OR #37 OR #38 OR #39 OR #40 OR #41 OR #42 OR #43 OR #44 OR #45 OR #46		6,347,182
48	#12 AND #47		27,764
49	France	ALL=(France)	
50	French	ALL=(France)	
51	france	(TI=(france)) OR AB=(france) OR OG=(france))	
52	french	(TI=(french)) OR AB=(french) OR OG=(french))	
53	#49 OR #50 OR #51 OR #52		4,589,169
54	#48 OR #53		2441
55	2022 or 2021 or 2020 or 2019 or 2018 or 2017 or 2016 or 2015 or 2014 or 2013 or 2012 or 2011 or 2010 or 2009 or 2008 or 2007 or 2006 or 2005 or 2004 or 2003 or 2002 or 2001 or 2000 (Publication Years) and English or French (Languages)		
**56**	**#54 AND #55**		**1949**

Table S4. Overview of study characteristics and results of included publications

Publication	Study design	Study population	Study date	Type of endpoint	Endpoint definition	Results and/or main findings
Aguilera<br>Emerging Infectious Diseases<br>2002<br>[52]	Cohort study	24 Hajj Piligrims and non piligrims diagnosed with IMD caused by serogroup W135	March-July 2000	IMD incidence	Incidence per population	▪ 21/100,000 inhabitants
					Proportion of IMD cases by age	▪ <1 year: 13% (n=3)<br>▪ 1–4: 29% (n=7)<br>▪ 5–9: 8% (n=2)<br>▪ 10–19: 8% (n=2)<br>▪ 20–49: 13% (n=3)<br>▪ 50–65: 17% (n=4)<br>▪ >65: 13% (n=3)
				Mortality	CRF	▪ 4/24 death reported (16.7%)<br>▪ Most of death occurred among patient aged > 20y
Antignac<br>Clinical Infect Dis<br>2003<br>[72]	Cohort study	2,167 clinical isolates of N. meningitidis from invasive infections	1999-2002	IMD incidence	Annual IMD incidence	▪ The annual incidence of culture-confirmed cases in France during the period of 1999–2002 was <1 case per 100,000 inhabitants.
					Distribution by serogroup	▪ Sg B: 58% (n=300 en 1999, n=342 en 2000, n=300 en 2001, n=297 en 2002)<br>▪ Sg C: 29% (n=103 en 1999, n=105 en 2000, n=204 en 2001, n=224 en 2002)<br>▪ Sg W: 8% (n=20 en 1999, n=59 en 2000, n=43 en 2001, n=58 en 2002)<br>▪ Sg Y: NR
					Distribution of strain by serotype	▪ Invasive meningococcal isolates were mainly nontypeable (40.4%)<br>▪ 29,2% of Sg B strains had a serotype 4<br>▪ 53% of Sg C strains had a serotype 2a and 27% had a serotype 2b<br>▪ 46,7% of Sg W135 strains had a serotype 2a
Arlet<br>Presse Médicale<br>2010	Case report	1 patient aged of 58 y admitted in Paris region	2010	Clinical presentation	Clinical presentation	▪ Endophthalmitis and arthritis secondary to meningococcal meningitis
				IMD incidence	Causing serogroup	▪ Sg C

Publication	Study design	Study population	Study date	Type of endpoint	Endpoint definition	Results and/or main findings
[54]				Long term sequelae	Type of sequelae according to the time follow-up	▪ 2 months after the discharge, persistent gonalgias.<br>▪ 5 months after the discharge, mechanical arthralgias.
Aubert<br>EuroCohort<br>2015<br>[73]	Case report	56 Sg C IMD cases reported in the Paris region	2013-2014	IMD incidence in June 2013	Number of cases in June 2013	▪ N=20 (including 3 cases in MSM)
					Distribution by genotype	▪ 3 isolates belonged to C:P1.5-1,10-8:F:3-6:cc11 (clonal complexe cc11)
				IMD incidence in July 2013-December 2014	Number of cases	▪ N= 36 (including 10 cases in MSM)
					Proportion of IMD cases by age	▪ 25 to 59y: 47% (n=17)<br>▪ 60 years and older: 14% (n=5)
					Distribution of strains by genotype	▪ 14/29 isolates related to the genotype C:P1.5-1,10-8:F3-6:cc11.<br>▪ Increase of IMD caused by C:P1.5-1,10-8:F3-6:cc11 isolates since 2011 particularly in the Paris region.
					Mean annual incidence of Sg C per population (compared to another French region)	▪ 0,05/100,000 inhabitants in Paris region (vs 0,02/100,000 elsewhere in France) from 2011 to 2014<br>▪ 2,28/100,000 inhabitants aged 25 to 59y being part of MSM community. The observed number of cases was 10 times greater than the expected number among men in this age group
				Mortality	CRF	▪ Between July 2013 and December 2014, 17% of reported cases died (n=6/36)
Barret<br>Médecine et Maladie infectieuse<br>2020<br>[74]	Case report	3 Sg W IMD cases reported in a Campus in Dijon	2016	IMD incidence	IMD incidence	▪ 10,8/100,000 inhabitants in a Campus in Dijon
					Distribution of strain by genotype	▪ W:P1.5,2:F1-1:cc11(UK-2013 strain): 100%
				Clinical presentation	Proportion of patient with certain presentation	▪ Septic shock: 2 (66%)<br>▪ Septic arthritis: 1 (34%)

Publication	Study design	Study population	Study date	Type of endpoint	Endpoint definition	Results and/or main findings
				Mortality	CRF	▪ 66% of reported cases died (n=2/3)
Bassi<br>EuroCohort<br>2017<br>[41]	Case report	2 Sg W IMD cases reported in a Campus in Paris	2017	IMD incidence	Distribution of strain by genotype	▪ W:P1.5,2:F1–1:cc11 (clonal complex cc11): 100%
				Clinical presentation	Distribution of patient with certain presentation	▪ Purpura fulminans + septic shock: 1 (50%)<br>▪ Meningitis with gastrointestinal pain (atypical presentation): 1 (50%)
				Mortality	CRF	▪ 50% of reported cases died (n=1/2)
Bilal<br>Pediatric Infectious Disease<br>2016<br>[37]	Cohort study	831 neonatal bacterial meningitis (<28 days of age) including 23 cases with confirmed meningococcal meningitis	2001-2013	IMD Incidence	Proportion of N.m infections in patient with bacterial meningitis	▪ 2,8% (n=23/831)
					Proportion of N.m cases by serogroup	▪ Sg B: 78% (n=18)<br>Sg C: 13% (n=3)<br>Sg others: 9% (n=2)
					Distribution by strain phenotype	▪ 33% of Sg C cases (n=1 case) belonged to C2a: P1.5,2<br>▪ 83% of Sg B cases (n=15) belonged to 3 NT:P1.4, 2 1:P1.14
				Clinical presentation	Proportion of patient with extensive purpura	▪ 9,1% of patients with meningococcal meningitis had an extensive purpura.
				Mortality	CRF	▪ 8,6% (n=2/23)
Bingen<br>Clinical Infectious Diseases<br>2005<br>[51]	Cohort study	1,084 children with bacterial meningitis (including 599 meningococcal cases)	2001-2003	IMD incidence	Proportion of N.m infections in patient with bacterial meningitis	▪ 55,3% (n=599/1084)
					Proportion of cases by serogroup	▪ Sg B: 58,3% (n=310/532)<br>▪ Sg C: 38,1% (n=203/532)<br>▪ Sg Other: 16,2% (n=86/532)
					Proportion of cases by age	▪ 1-2m<: 2% (n=12)<br>▪ 2-12m<: 24,9% (n=149)<br>▪ 1-2y<: 15,3% (n=92)<br>▪ 2-15y<: 53,7% (n=322)<br>▪ 15-18y: 4% (n=24)

Publication	Study design	Study population	Study date	Type of endpoint	Endpoint definition	Results and/or main findings
					Proportion of Sg C by year	▪ 2001: 38.1%<br>▪ 2002: 42.3%<br>▪ 2003: 34.6%
				Mortality	CRF	▪ 7.6%. of reported cases died
					Distribution by serogroup	▪ Sg B: 8,2%<br>▪ Sg C: 8,4%
Briand<br>Médecine et maladies infectieuses<br>2016<br>[55]	Cohort study	34 children with bacterial meningitis (including 18 children with meningococcal meningitis) in Paris area (Bondy)	2001-2013	IMD incidence	Proportion of N.m infections in patient with bacterial meningitis	▪ 53% (n=18/34)
					Distribution by serogroup	▪ Sg B: 61% (n=11)<br>▪ Sg C: 17% (n=3)<br>▪ Sg W: 11% (n=2)
Caron<br>Lancet Infectious Disease<br>2011<br>[24]	Cohort study	149 cases of IMD reported in Seine-Maritime (1990-2001)	1990-2010	IMD incidence in 1990-2001	Proportion cases with Sg B	▪ 85% (n=118/149)
					Distribution by strain phenotype	▪ 34% of Sg B cases (n=37/108) belongs to B:14:P1.7,16
		134 cases of IMD reported in Seine-Maritime (2003-2006)		IMD incidence in 2003-2006	Proportion of Sg B cases	▪ 80% (n=91/114)
					Distribution by strain phenotype	▪ 66% of Sg B cases belongs to B:14:P1.7,16 (n=47/71)
					Incidence rate of B:14:P1.7,16 per population	▪ 11,4/100 000 inhabitants in Dieppe area<br>▪ 1,2/100 000 inhabitants in Seine Maritime
				Mortality	CRF	▪ 19% of confirmed Sg B cases died (n=9) and 5% of probable cases died (n=2) in Seine Maritime in 1990-2001<br>▪ 25% of confirmed and probable Sg B cases died (n=6) in Seine Maritime in 2003 (vs 5% in 2004 and 10% in 2005)
Cohen<br>Archives de Pédiatrie<br>2003<br>[75]	Cohort study	449 children with bacterial meningitis (including 194 children with meningococcal meningitis)	2001	IMD incidence	Proportion of N.m infections in patient with bacterial meningitis	▪ 43,2% (n=194/449)
					Proportion of N.m cases by age	▪ < 1 m: 4,4% (n=4)<br>▪ 1-3m: 24,2%(n=8)<br>▪ 3-24 m: 41,6% (n=72)<br>▪ > 24 m: 56,7% (n=110)
				Mortality	CRF	▪ 5,1% of reported cases died (n=10)

Publication	Study design	Study population	Study date	Type of endpoint	Endpoint definition	Results and/or main findings
Contou<br>Intensive Care Med<br>2018<br>[47]	Cohort study	306 adult patients with purpura fulminans (including 195 cases caused by N.m)	2000-2016	IMD incidence	Proportion of N.m infections in patients with purpura fulminans	▪ 63,7% (n=195/306)
					Distribution by serogroup	▪ Sg B: 39%<br>▪ Sg C: 34%
				Long term sequelae	Proportion of patient without amputation	▪ 51,3% were not amputated (N=100/195)
					Proportion of patient with amputation	▪ 11,8% had an amputation (n=23/195)
				Mortality	CRF	▪ 36,9% of reported cases died (n=72/195)
Deghmane<br>The journal of infectious diseases<br>2010<br>[18]	Cohort study	6,528 IMD cases reported to the Institut de Veille sanitaire	1999-2008	IMD incidence	Number of IMD cases	▪ N=6528 cases were reported
					Incidence rate by year (corrected by underreporting)	▪ 1999: 0,98/100,000 inhabitants<br>2002: 1,43/100,000 inhabitants<br>2003: 1,61/100,000 inhabitants<br>2008: 1,2/100,000 inhabitants
					Proportion of IMD cases by serogroup	▪ N= 5763 of cases with known serogroup<br>▪ Sg B: 62% (n=3564)<br>▪ Sg C: 29% (n=1677)<br>▪ SgW135: 5% (n=274)<br>▪ Sg Y: 3% (n=170)<br>▪ The peak of Men C observed in 2002 was mainly due to isolates of phenotype C:2a:P1.5 of the sequence type 11 (ST-11) clonal complex.<br>▪ Appearance of a new phenotype, C:2a:P1.7,1 of the sequence type 11 (ST-11) clonal complex in 2003 (even if there is a decrease in the number of Sg C since 2003)
					Incidence of Sg C (1999-2022)	▪ 0,5/100,000 inhabitants<br>▪ Proportion of Sg C : 41%
					Proportion of isolates with C:2a:P1.7,1 phenotype	▪ N= 81 isolates belong to C:2a:P1.7,1 since 1999 (90% have been isolated since 2005)
					Proportion of C:2a:P1.7,1 strain by year	▪ 2005: 6.6%<br>2006: 10.3%<br>2007: 21.9%<br>2008: 24.5%

Publication	Study design	Study population	Study date	Type of endpoint	Endpoint definition	Results and/or main findings
				Clinical presentation	Proportion of patient with certain presentation	▪ 42% (n=30/71) infected by C:2a:P1.7,1 had extensive hemorrhagic rash, been statically higher than for cases with other isolates.
				Mortality	CRF	▪ 22% of patients with isolate C:2a:P1.7,1 died (vs 15% for cases of infection with other serogroup C isolates)
Deghmane<br>J Infect<br>2020<br>[42]	Cohort study	27 IMD cases due to ST-9316 lineage	2013-2018	IMD incidence at <u>national level</u>	Number of IMD cases due to ST-9316	▪ N=27
					Proportion of IMD ST-9316 cases by serogroup	▪ Sg B: 22% (n=6)<br>▪ Sg C: 4% (n=1)<br>▪ Sg W: 74% (n=10)
					Distribution of IMD ST-9316 cases by year	▪ 2013: 1<br>2015: 2<br>2016: 6<br>2017: 9<br>2018: 9<br>▪ 70% of IMD cases belonging to ST-9316 were residents of the Hauts de France.<br>▪
					Distribution of IMD cases with Sg <u>W</u> ST-9316 by year	2013: 1<br>2015: 2<br>2016: 6<br>2017: 6<br>2018: 5
					Incidence rate of Sg W by year per population	▪ 0,04/100,000 inhabitants in 2013 to 0,09/100,00 inhabitants in 2018<br>▪ This increase was mainly due to the expansion of isolates belonging to the South American/UK sublineage
				IMD incidence <u>In Haut de France</u>	Number of Sg W cases	▪ N=29 (vs 230 in the rest of France)<br>▪ 52% belonged to ST-9316 (vs 2% for the rest of the country)
					Incidence rate of Sg W by year per population	▪ 0,05/100,000 inhabitants in 2013 to 0,12/100,00 inhabitants in 2018<br>▪ 52% (n = 15/29) belonged to ST-9316 in Hauts-de-France versus 2%, (n = 5/230) for the rest of the country.

Publication	Study design	Study population	Study date	Type of endpoint	Endpoint definition	Results and/or main findings
					Distribution by age of IMD W ST-9316	▪ < 5y: 67% (vs 9% in patient with SgW/CC11)<br>6-14 y: 0% (vs 0% in patient with SgW/CC11)<br>15-24 y: 0% (vs 18% in patient with SgW/CC11)<br>25-49y: 7% (vs 9% in patient with SgW/CC11)<br>≥ 50 y: 27% (vs 55% in patient with SgW/CC11)
				Clinical presentation	Distribution of patient with septic choc including purpura fulminans by complex clonal	▪ 13% of cases with Sg W/ ST9317 had a septic choc in Haut de France<br>▪ 73% of cases with Sg W/cc11 had a septic choc in Haut de France
				Mortality	Case fatality rate in patient with IMD W/ST-9316 in Haut-de-France	▪ 6,6% of reported cases died (vs 33% in patient with Sg W/cc11) in Haut de France<br>▪ Sg W/ST-9316 showed lower virulence in mice compared to Sg W/cc11 isolates.
				Long-term sequelae	Proportion of patient with sequelae	▪ 0% of patient with IMD W/ST-9316 (vs 9% in patient with IMD W/CC11) in Haut de France
Deghmane Microorganisms 2022 [20]	Cohort study	1,595 clinical bacterial isolates of N.m	2017-2021	IMD incidence	Number of IMD cases by year	▪ 2017: 474<br>▪ 2018: 397<br>▪ 2019: 416<br>▪ 2020: 202<br>▪ 2021: 106
					Distribution by serogroup	▪ Sg B: 49,6% (n=791)<br>▪ Sg C: 17,9% (n=286)<br>▪ SgW135: 17% (n=271)<br>▪ Sg Y: 13,9% (n=221)<br>▪ The yearly distribution of the serogroups over the studied 5-year period showed decreasing numbers of cases since the declaration of the COVID-19 pandemic in comparison to the period prior to this pandemic, although the decrease seemed to be less prominent in 2021 compared to 2020.<br>▪ Serogroup B remains the most frequent serogroup in all age groups (except for 65 years and older).<br>▪ The most prominent trend was the decreasing proportion of isolates belonging to the

Publication	Study design	Study population	Study date	Type of endpoint	Endpoint definition	Results and/or main findings
						hyperinvasive clonal complexes that was mainly due to the decrease in isolates of CC11.
Delisle 2010 Euro Cohort [23]	Case report	11 cases of IMD group B notified in Landes, Aquitaine region (aged between 7m to 47y)	2008-2009	IMD incidence	Incidence rate of Sg B cases	▪ 3,0/100,000 inhabitants in Landes (n=11)<br>▪ 8,9/100,000 inhabitants in Dax area (n=8)<br>▪ 0,6/100,000 inhabitants at a national level (n=414)
					Proportion of Sg B cases by age in Landes (vs in the rest of France)	▪ <5y: 27% (n=3) (vs 46% (n=189))<br>5-14y: 9% (n=1) (vs n=10% (n=43))<br>15-19y: 18% (n=2) (vs 15% n=63))<br>20-24y: 36% (n=4) (vs 10% (n=42))<br>≥25y: 9% (n=1) (vs 19% (n=77))
					Distribution by strain phenotype	▪ 10% of the strains responsible for IMD analyzed by the CNR in 2009 belonged to CC ST-269 (5% in the last ten years).<br>▪ These isolates may differ in virulence, as has been observed for the common CCs ST-32 and ST 41/44 and seem to be highly transmissible.
				Clinical presentation	Proportion of patient with certain presentation	▪ 18% (n=2) of cases presented with purpura fulminans
				Mortality	CRF	▪ 9% of patients died (n=1/11), presenting a purpura fulminans
Dubos 2009 Arch Pediat [33]	Cohort study	319 IMD cases notified in PMSI data base and reported in the Institut de Veille Sanitaire (InVS) (aged between 0 to 17y) in Nord Pas de Calais region	2002-2005	IMD incidence	Corrected incidence rate by year	▪ 2002: 6,9/100,000 inhabitants<br>▪ 2003: 8,3/100,000 inhabitants<br>▪ 2004: 6,9/100,000 inhabitants<br>▪ 2005: 9,4/100,000 inhabitants
					Distribution of Sg B by age (2005)	▪ <1y: 33,4/100,000 inhabitants<br>▪ 1-4y: 8,3/100,000 inhabitants<br>▪ 5-17y: 1,4/100,000 inhabitants

Publication	Study design	Study population	Study date	Type of endpoint	Endpoint definition	Results and/or main findings
					Distribution by serogroup	▪ Sg B: 56%<br>▪ Sg C: 16%<br>▪ Sg A, Y or W135: 3%
				Clinical presentation	Proportion of IMD cases by clinical presentation	▪ Meningococcemia without septic shock: 12%<br>▪ Meningitis: 58%<br>▪ Purpura fulminans: 28%<br>▪
				Mortality	CRF	▪ 9,3% of reported cases died
Duval<br>Advances in Therapy<br>2022<br>[40]	Cohort study	111 adult patients who had been diagnosed with meningococcal meningitis with or without purpura fulminans	February 2013- July 2015	Clinical presentation	Distribution of IMD cases by clinical presentation	▪ Meningitis without purpura fulminans (PF): 63,1% (n=70)<br>▪ Meningitis with PF: 36,9% (n=41)
				IMD incidence	Distribution of IMD cases by serogroup	▪ N=69 patients with known serogroup and follow-up at 12m<br>▪ Sg B: 52% (n=36)<br>▪ Sg C: 39% (n=27)<br>▪ Sg Y: 9% (n=6)
				Mortality	CRF	▪ 4,5% of included patients died
				Long term sequelae	Proportion of patient with certain long-term sequelae	▪ N=71 patients with follow-up at 12m<br>▪ Persistent headache: 32,9% (n=23/70)<br>▪ Dissatisfied or very dissatisfied with sleep: 42,9% (30/70)<br>▪ Not at all able to focus: 10% (7/70)<br>▪ Depressive symptoms: 34,3% (24/70)<br>▪ Hearing loss: 15,5% (11/71)
Faye<br>Archives de Pédiatrie<br>2005<br>[32]	Case report	5 children reported cases of IMD due to Sg W135 in Paris region between june 2000 and december 2022 (aged between 19m and 11y)	2000-2002	Clinical presentation	Proportion of patient with certain clinical presentation	▪ Meningitis: 60% (n=3/5)<br>▪ Arthritis: 40% (n=2/5)
				Long-term sequelae	Number of patients with or without long-term sequelae	▪ Patients were followed for an average of 18 months [5–22 m].<br>▪ No sequelae: 4/5<br>▪ At least 1 sequela: 1/5<br>▪ A persisted inflammatory syndrome
Floret<br>Arch Pediat<br>2001	Cohort study	100 children aged from 10 days to 18y died from community acquired bacterial	1999-2000	Mortality	Proportion of N.m infections in patient with bacterial meningitis	▪ 24% (n=24) died following N.m infection

Publication	Study design	Study population	Study date	Type of endpoint	Endpoint definition	Results and/or main findings
[35]		infections (including 24 cases caused by N.m)			Distribution by serogroup	▪ Sg B: 58% (n=14/24)<br>Sg C: 25% (n=6/24)<br>Sg W135: 8% (n=2/24)
					Distribution by age	▪ <2m: 12,5% (n=3/24)<br>▪ > 2m: 87,5% (n=21/24)
					Distribution by clinical presentation	▪ Meningitis: 21% (n=5/24)<br>▪ Sg B: 60% (n=3/5)<br>▪ Sg W135: 40% (n=2/5)<br>▪ Purpura fulminans: 79% (n=19/24)<br>▪ Sg B: 57,9% (n=11/19)<br>▪ Sg C: 31,6% (n=6/19)
Garnier<br>Diagn Microbiol Infect Dis<br>2011<br>[76]	Cohort study	21 IMD cases with Sg C aged from 3m to 70y in Haute-Vienne County	2003-2007	IMD incidence	Distribution by clonal complex	▪ 20/21 isolates belonged to the ST-11 clonal complex (8 to clone ET-15 and 12 to clone ET-37) and 1 to the ST-8 clonal complex<br>▪ Between August 2004 and June 2005, and again between April 2006 and March 2007, the incidence rate of serogroup C meningococcal disease in the Haute-Vienne County of France was almost 8 times than the overall French average.
				Clinical presentation	Proportion of patient with purpura fulminans	▪ 28% (n=6/21)
				Mortality	CRF	▪ 9% of reported patients died
				Long term sequelae	Number of patients with sequelae	▪ N=4/21 had major sequelae following IMD
Gaschignard<br>Pediatr Infect Dis J<br>2013<br>[34]	Cohort study	119 IMD cases with Sg W aged from 0 to 17y	2001-2008	Clinical presentation	Distribution by clinical presentation	▪ Meningitis: 66% (n=78/119)<br>▪ Meningococcemia only: 21% (n=25/119)<br>▪ Septic arthrisis: 8% (n=10/119)<br>▪ Purpura fulminans: 5% (n=6/119)
				IMD incidence	Distribution by age	▪ The highest number of patients was in infants between 6 and 9m

Publication	Study design	Study population	Study date	Type of endpoint	Endpoint definition	Results and/or main findings
					Distribution of strain clonal complex	▪ ST-22: 50%<br>▪ ST-11: 41% (hyperinvasive clonal complexes)<br>▪ Other ST: 9%
				Mortality	CRF	• 6% of reported patients died (n=7/119)
Grodet<br>Clinical Microbiology and Infection<br>2004<br>[77]	Case report	8 IMD cases with Sg B aged between 14 and 28y in Indre-et-Loire region	2000-2002	IMD incidence	Distribution by strain phenotype	▪ 8 isolates were caused by N. meningitidis B:15:P1.12 and belonged to ST-1403
				Mortality	CRF	▪ 12,5% of reported cases died
				Long term sequelae	Number of patients with sequelae	▪ N=2/8 patient presented at least on minor sequelae<br>▪ Diplopia: 2<br>▪ Vertigo: 1<br>▪ N= 0 patient presented major neurological sequelae
					Distribution by strain clonal complex for Sg B isolates	▪ CC41/44: 29,8% in 2013-2014 (vs 13,3% in 2018-2019)<br>CC32: 27,5% in 2013-2014 (vs 34,8% in in 2018-2019)<br>▪ Hyperinvasive CC (including CC11): 67,8% in 2013-2014 (vs no cc11 in 2018-2019)
Hong<br>J Infect<br>2018<br>[30]	Cohort study	527 samples from IMD cases of Sg W	2000-2016	IMD incidence	Distribution by age group	▪ N= 77 Sg W cases in 2015-2016<br>▪ 0-14y: 19,5% (n=15) (vs 40,7% in Sg BCY)<br>▪ 15-24y: 27,3% (n=21) (vs 20% in Sg BCY)<br>▪ 25-59: 27,3% (n=21) (vs 21,6% in Sg BCY)<br>▪ >60y: 26% (n=20) (vs 17,8% in Sg BCY)<br>▪ The proportion of cases in age groups above 15 years old was higher for group W compared to group B and C cases in 2015-2016.
					Proportion of Sg W isolates with certain complex clonal	▪ cc11: 53% (n=195)<br>▪ cc22: 35% (n=128)<br>▪ Others cc: 12% (n=43)<br>▪ The cc11 isolates were responsible for the increase of NmW IMD observed at the beginning of the 2000s, in 2012 and in 2016<br>▪ An increase in IMD was observed in 2012 and was linked to isolates belonging to the "Anglo-

Publication	Study design	Study population	Study date	Type of endpoint	Endpoint definition	Results and/or main findings
						French-Hajj" sub-lineage. These isolates have decreased significantly since 2013 and have been replaced by NmW/cc11 isolates related to the "South American - UK" sub-lineage which caused a marked increase in the number of cases of NmW in 2016.<br>▪ A changing pattern in the epidemiology of NmW has been observed in 2015-2016 in relation to the spread of the "UK 2013-strain".
				Clinical presentation	Proportion of patient with certain clinical presentation	▪ Meningitis: 57,1% (vs 74% in Sg BCY)<br>▪ Septicemia: 77,9% (vs 56,9% in Sg BCY)<br>▪ Arthritis: 9,1% (vs 3,6% in Sg BCY)<br>▪ Purpura fulminans: 13,1% (vs 22,7% in Sg BCY)
				Mortality	Distribution by serogroup	▪ Sg B: 7,8%<br>▪ Sg C: 12,3%<br>▪ Sg W: 22.1%<br>▪ Sg Y: 17,2%
					Distribution by sub-lineage (Sg W)	▪ Original UK strain: 22,7%<br>▪ UK 2013 strain: 27,8%<br>▪ Anglo-French Hajj: 4,0%<br>▪ Non-cc11 lineages: 15,9%
Hong<br>Human Vaccines and Immunotherapeutics<br>2021<br>[78]	Cohort study	428 cases of IMD confirmed between 2013 and 2014<br>366 cases of IMD confirmed between 2018 and 2019	July 2013-June 2014<br>July 2018-June 2019	IMD incidence	Proportion of Sg B cases by year	▪ 2013-2014: 55%<br>2018-2019: 51,4%
					Distribution by strain clonal complex for Sg B isolates	▪ CC41/44: 29,8% in 2013-2014 (vs 13,3% in 2018-2019)<br>CC32: 27,5% in 2013-2014 (vs 34,8% in in 2018-2019)<br>▪ Hyperinvasive CC (including CC11): 67,8% in 2013-2014 (vs no cc11 in 2018-2019)
Huang<br>Plos One<br>2022<br>[43]	Cohort study	1,344 IMD cases notified in PMSI data base	2014-2016	IMD incidence	Distribution by age group	▪ <1y: 15% (n=208)<br>▪ 1-4y: 14% (n=192)<br>▪ 5-14y: 11% (n=149)<br>▪ 15-24y: 13% (n=171)<br>▪ 25-59y: 17% (n=231)

Publication	Study design	Study population	Study date	Type of endpoint	Endpoint definition	Results and/or main findings
						▪ ≥60y: 14% (n=203)
				Clinical presentation	Proportion of cases with certain clinical presentation	▪ Meningitis: 58,8% (n=790)<br>▪ Septicemia: 25,1% (n=338)<br>▪ Meningitis + septicemia: 9,4% (n=127)<br>▪ Other type of IMD: 6,6% (n=89)
					Distribution by age	<u>Children (0-19y)</u><br>▪ Meningitis: 61,1%<br>▪ Septicemia: 22,2%<br>▪ Meningitis + septicemia: 12,3%<br>▪ Other type of IMD: 4,5%<br><u>Adults (>20y)</u><br>▪ Meningitis: 56,5%<br>▪ Septicemia: 28,2%<br>▪ Meningitis + septicemia: 6,5%<br>▪ Other type of IMD: 8,8%
				Mortality	CRF	▪ 6.0% of reported cases died
					Distribution by clinical presentation	▪ According to clinical presentation:<br>▪ Meningitis: 5,6%<br>▪ Septicemia: 7,7%<br>▪ Meningitis + septicemia: 3,9%
				Long term sequelae	Proportion of patient with certain sequelae	▪ 13,6% (n = 183) had at least one sequela at index hospital discharge<br>▪ 19,4% (n=261) had at least one sequela during the entire study<br>▪ Type of sequelae:<br>▪ Neurological sequelae: 11,5% (n = 154)<br>Auditive impairment: 2,8% (n=37)<br>Amputation or skin necrosis or skin grafting: 2,7% (n = 36)<br>Cognitive impairment: 1,7% (n=23)<br>Chronic renal failure: 1,2% (n=16)<br>Arthritis: 0,7% (n=10)<br>▪ About 30% of sequelae were developed after discharge from the index hospitalization.

Publication	Study design	Study population	Study date	Type of endpoint	Endpoint definition	Results and/or main findings
						▪ Notably, about 70% of cognitive impairment was diagnosed during the follow-up period (n=16/23)
					Distribution by clinical presentation	▪ The highest proportion of patients with at least one sequela during the entire study period was observed in patients with both septicemia and meningitis (21,3%), followed by patients with meningitis only (19,9%) and septicemia only (18,3%).
				Hospitalization details	Hospitalization details	▪ Mean Length of hospitalization: 12 days (median: 9 days; IQR:7-13 days)<br>▪ ICU admission: 44,9%<br>▪ Discharge status:<br>▪ Rehabilitation center: 5%<br>▪ Home: 79,1%<br>▪ Other: 10%
Levy<br>Arch Pediat<br>2008<br>[39]	Cohort study	2,951 children diagnosed with bacterial meningitis (including 1,344 cases of meningococcal meningitis)	2001-2007	IMD incidence	Proportion of N.m infections in patient with bacterial meningitis	▪ 46% (n=1,344)
					Number of cases by year	▪ 2001: 200<br>2002: 189<br>2003: 230<br>2004: 157<br>2005: 195<br>2006: 206<br>2007: 167
					Distribution by serogroup and by year	▪ Sg B: 59% (n=796)<br>2001: 104<br>2002: 90<br>2003: 131<br>2004: 101<br>2005: 124<br>2006: 133<br>2007: 113<br><br>Sg C: 29% (n=389)<br><br>▪ Sg W135: 2,4% (n=32)<br>▪ 2001: 3<br>▪ 2002: 8<br>▪ 2003: 7<br>▪ 2004: 4<br>▪ 2005: 4<br>▪ 2006: 6<br>▪ 2007: 0<br><br>Sg Y: 0,7% (n=9)

Publication	Study design	Study population	Study date	Type of endpoint	Endpoint definition	Results and/or main findings	
						2001: 67<br>2002: 76<br>2003: 72<br>2004: 40<br>2005: 43<br>2006: 50<br>2007: 41	▪ 2001: 2<br>▪ 2002: 1<br>▪ 2003: 2<br>▪ 2004: 0<br>▪ 2005: 0<br>▪ 2006: 1<br>▪ 2007: 3
				Clinical presentation	Proportion of patient with extensive purpura	▪ 29,5% of cases had an extensive purpura	
				Mortality	CRF	▪ 6,6% of reported patient died	
					Distribution by serogroup	▪ Sg B: 5,5%<br>Sg C: 9,9%	
Levy<br>Archives de Pédiatrie<br>2008<br>[45]	Cohort study	2,951 children diagnosed with bacterial meningitis (including 1,344 cases of meningococcal meningitis)	2001-2007	IMD incidence	Proportion of N.m infections in patient with bacterial meningitis	▪ 46% (n=1,344)	
					Number of cases by year	▪ 2001: 200<br>2002: 189<br>2003: 230<br>2004: 157<br>2005: 195<br>2006: 206<br>2007: 167	
					Number of cases by serogroup and by year	▪ Sg B: 59% (n=796)<br>2001: 104<br>2002: 90<br>2003: 131<br>2004: 101<br>2005: 124<br>2006: 133<br>2007: 113	Sg C: 29% (n=389)<br>2001: 67<br>2002: 76<br>2003: 72<br>2004: 40<br>2005: 43<br>2006: 50<br>▪ 2007: 41
					Distribution by age group	▪ < 1m: 1,0% (n=14)<br>▪ 1-2m: 2,0% (n=27)<br>▪ 2-12m: 26,9% (n=361)<br>▪ 12-24m: 13,7% (n=184)<br>▪ 24m-5y: 24,8% (n=334)<br>▪ > 5y: 31,5% (n=424)	
				Mortality	CRF	▪ 6,6% of reported cases died	

Publication	Study design	Study population	Study date	Type of endpoint	Endpoint definition	Results and/or main findings
Levy<br>Pediat Infect Dis<br>2010<br>[38]	Cohort study	2,131 children diagnosed with bacterial meningitis (including 962 cases of meningococcal meningitis)	2001-2005	IMD incidence	Proportion of N.m infections in patient with bacterial meningitis	▪ 45% (n=962)
					Distribution by serogroup	▪ Sg B: 62,3%<br>▪ Sg C: 33,7%<br>▪ Sg W135: 2,9%<br>▪ Sg Y: 0,6%
					Distribution by clonal complex	▪ ST 41/44: 32,2%<br>▪ ST 11: 21,9%<br>▪ ST 32: 20,8%<br>▪ ST 8: 8,2%<br>▪ ST 269: 4,9%<br>▪ ST-32 and ST-269 were associated with serogroup B (>96.6%) and ST-11 and ST-8 were associated with serogroup C (>75%).<br><br>▪ The geographic heterogeneity of distribution was marked by spatiotemporal localization such as phenotype B:14:P1.7,16 isolates tended to cluster in the haute Normandie region (56%, P<0.001).
					Distribution by age group	▪ 1d-<1y: 27,8% (n=267)<br>▪ ≥1y->5y: 39,9% (n=384)<br>▪ ≥5-<10y: 18,9% (n=182)<br>▪ ≥10-18y: 13,4% (n=129)
				Clinical presentation	Proportion of patient with extensive purpura	▪ 29,7% of cases had an extensive purpura
				Mortality	CRF	▪ 6,9% of reported cases died
					Distribution by age	▪ 1d-<1y: 8% (n=21)<br>≥1y-<5y: 8,4% (n=32)<br>≥5-<10y: 5,5% (n=10)<br>≥10-18y: 2,3% (n=3)
					Distribution by serogroup	▪ Sg B: 5,9%<br>▪ Sg C: 9,9%

Publication	Study design	Study population	Study date	Type of endpoint	Endpoint definition	Results and/or main findings
						▪ Children with phenotype B:14:P1.7,16 isolates tended to be older (8.6 years) with a higher case fatality rate (12%, P=0.04).<br><br>▪ The phenotype/genotype C:2a:P1.5/ST-11 was found in 26.3% of serogroup C cases and was possibly associated with a higher mortality among serogroup C (9.9% for C and 5.9% for B, P = 0.04).
Levy<br>Archi Pediat<br>2012<br>[49]	Cohort study	3,769 children diagnosed with bacterial meningitis (including 1,661 cases with meningococcal meningitis)	2001-2009	IMD incidence	Proportion of N.m infections in patient with bacterial meningitis	▪ 44,1% (n=1,661)
					Distribution by serogroup	▪ Sg B: 61,3%<br>▪ Sg C: 27,0%<br>▪ Sg W135: 2,4%<br>▪ Sg Y: 0,6%
				Clinical presentation	Distribution by serogroup	▪ 29,8% of cases with Sg B had a purpura fulminans<br>▪ 29,5% of cases with Sg C had a purpura fulminans
				Mortality	CRF	▪ 6,5% of reported cases died
					Distribution by serogroup	▪ Sg B: 5,9%<br>▪ Sg C: 9,2%
Lévy<br>Achives de Pédiatrie<br>2014<br>[50]	Cohort study	4,808 children diagnosed with bacterial meningitis (including 1,991 cases of meningococcal meningitis)	2001-2012	IMD incidence	Proportion of N.m infections in patient with bacterial meningitis	▪ 41,4% (n=1,991)
					Number of cases by year	2001: 200<br>2002: 189<br>2003: 230<br>2004: 159<br>2005: 196<br>2006: 206<br>2007: 173<br>2008: 174

Publication	Study design	Study population	Study date	Type of endpoint	Endpoint definition	Results and/or main findings
						2009: 138<br>2010: 110<br>2011: 109<br>2012: 107
					Proportion of cases by serogroup and by year	Sg B = 64%<br>2001: 52% (n=104)<br>2002: 47,9% (n=90)<br>2003: 57 (n=131)<br>2004: 64,2% (n=102)<br>2005: 63,3% (n=124)<br>2006: 64,9% (n=133)<br>2007: 68,2% (n=118)<br>2008: 69,9% (n=121)<br>2009: 69,6% (n=96)<br>2010: 71,8% (n=79)<br>2011: 79,8% (n=87)<br>2012: 80,4% (n=86)<br><br>Sg C = 24%<br>2001: 33,5% (n=67)<br>2002: 40,2% (n=76)<br>2003: 31,3% (n=72)<br>2004: 25,8% (n=41)<br>2005: 22,5% (n=44)<br>2006: 23,9% (n=49)<br>2007: 24,3% (n=42)<br>2008: 20,2% (n=36)<br>2009: 17,4% (n=24)<br>2010: 15,5% (n=17)<br>2011: 10,1% (n=11)<br>2012: 8,4% (n=9)<br><br>▪ Number of Sg C cases decreased by 87% between 2001 and 2012 (n=67 to n=9).
					Distribution by age group	▪ <1m: 0,9% (n=19)<br>▪ 1-2m: 2,4% (n=47)<br>▪ 2-12m: 27,7% (n=551)<br>▪ 12-24m: 14,6% (n=290)<br>▪ 24m-5y: 23,4% (n=467)<br>▪ >5y: 31,0% (n=617)
				Mortality	CRF	▪ 5,9% of reported cases died
					Distribution by serogroup	▪ Sg B: 5,3%<br>▪ Sg C: 8,9%
				Mortality	CRF	▪ 6,6% of reported cases died
Lévy-Bruhl EuroCohort 2002 [79]	Case report	17 confirmed IMD cases in the Puy-de-dôme department	2001-2002	IMD incidence	Distribution of cases by serogroup	▪ Sg B: 13% (n=2)<br>Sg C: 73% (n=11)
					Incidence rate of Sg C in 2001	▪ 1,7 cases/100 000 inhabitants in Pyu-de-Domes (vs 0,3/100 000 at national level)

Publication	Study design	Study population	Study date	Type of endpoint	Endpoint definition	Results and/or main findings
					Distribution by age group	▪ The distribution of the patients by age group did not differ from the one observed at national level (p=0.45), with six out of 11 cases (55%) in children below five years of age.
				Clinical presentation	Proportion of Sg C cases with purpura fulminans	▪ 64% (n=7/11) (vs 27% at national level)
				Mortality	CRF of Sg C cases with purpura fulminans	▪ 27% (n=3/11) (vs 15% at national level)<br>▪ One of the groups (5 strains) belonged to the ET-37 clonal complex, known to include epidemic strains, and was responsible for the deaths of the 3 IMD cases
Lévy-Bruhl EuroCohort 2019 [27]	Cohort study	2 cohorts of children born january to may 2017 and january to may 2018	2017-2018	IMD incidence	Number of Sg cases	▪ This sharp increase in MenC VC translated into a dramatic decrease in the number of invasive MenC disease cases notified in infants through the mandatory notification system, from 17 cases on average during the 2012–16 period to 4 in 2018, all in non-vaccinated individuals.<br>▪ This contrasts with the very limited decrease in incidence in individuals above 1 year of age in 2018.
Lorton Pediatric and perinatal epidemiology 2018 [53]	Cohort study	124 children (aged between 1m to 16y) diagnosed with pneumococcal or meningococcal infection (including 75 meningococcal cases) in two departments (Loire Atlantique and Vendée)	2009-2014	IMD incidence	Proportion of N.m infections in patient with bacterial meningitis	▪ 60% (n=75)
					Distribution by serogroup	▪ Sg B: 62,7% (n=47/75)<br>▪ Sg C: 20% (n=15/75)<br>▪ Sg W135: 1,3% (n=1/75)<br>▪ SG Y: 1,3% (n=1/75)
				Mortality	CRF	▪ 13,3% of reported cases died (n=10)
					Distribution by serogroup	▪ Sg B: 30% (n=3/10)<br>▪ Sg C: 50% (n=5/10)<br>▪ Sg W135: 0<br>▪ SG Y: 0

Publication	Study design	Study population	Study date	Type of endpoint	Endpoint definition	Results and/or main findings
				Long term sequelae	Number of patients with sequelae	▪ N=7/10 patient had severe sequelae at hospital discharge
					Distribution by serogroup	▪ Sg B: 42,8% (n=3/7)<br>▪ Sg C: 28,6% (n=2/7)<br>▪ Sg W135: 0<br>▪ Sg Y: 14,3% (n=1/7)<br>▪ Not available for 1 case
Lorton<br>Pediatr Crit Care Med<br>2020<br>[36]	Cohort study	261 children (aged between 1m to 16y) diagnosed with bacterial infection (including 75 meningococcal cases) in two departments (Loire Atlantique and Vendée)	2009-2014	IMD incidence	Proportion of N.m infections in patient with bacterial meningitis	▪ 29% (n=75)
					Incidence rate	▪ The incidence rates for COSBIs due to N. meningitidis decreased between 2000–2006 and 2009–2014, from 2.7 to 0.8 cases per 100 000 person-years (70% relative decrease; RR = 0.30; 95% CI, 0.22–0.44)
					Distribution by serogroup	▪ Sg B: 63%<br>▪ Sg C: 20%<br>▪ Sg W: 1%<br>▪ Sg Y: 1%
					Distribution by serogroup and by year for children older > 3m	▪ Sg B:<br>▪ 2000-2006: 29%<br>▪ 2009-2014: 44%<br><br>▪ Sg C:<br>▪ 2000-2006: 14%<br>▪ 2009-2014: 14%<br>▪
				Clinical presentation	Proportion of patient with certain clinical presentation	▪ Meningitis: 32,0% (n=24/75)<br>▪ Purpura fulminans: 64,0% (n=48/75)<br>▪ Sepsis: 2,7% (n=2/75)<br>▪ Pneumonia: 1,3% (n=1/75)
Parent du Chatelet<br>Archiv Pediat<br>2007<br>[25]	Cohort study	147 IMD cases in Seine Martime region (including 101 Sg B cases)	2003-2006	IMD incidence	Annual incidence rate by year	▪ 2003-2004: 2,4/100 000 in Seine Maritime (vs 1,6/ 100 000 at a national level)<br>▪ 2005-2006: 3,5/100 000 in Seine Maritime (vs 1,5/100 000 at a national level)

Publication	Study design	Study population	Study date	Type of endpoint	Endpoint definition	Results and/or main findings
					Distribution of Sg B cases by year	▪ 2003: 87%<br>▪ 2004: 82%<br>▪ 2005: 89%<br>▪ 2006: 65%<br>In 2006, the proportion of Sg B was lower due to the increase of Sg C
					Incidence rate of Sg B cases	▪ 2,7/100,000 (vs 0,8/100,000 at a national level) in 2003 and 2005
					Proportion of Sg B by year (cases aged <5y)	▪ Before 2002: 54%<br>▪ 2003-2006: 36%
					Proportion of B:14:P1.7,16 strain	▪ 65% of Sg B strain belonged to B:14:P1.7,16 strain (vs 5% at a national level)
					Annual incidence rate of B:14:P1.7,16 cases	▪ In Dieppe town: 11,5 / 100,000 (vs 1,2/100,000 at a national level)
					Distribution of B:14:P1.7,16 cases by age group	▪ In Seine-Maritime region, 2003-2006:<br>▪ <5y: 34%<br>▪ <20y: 84%<br><br>▪ In Dieppe town, 2003-2006:<br>▪ <1y: 46,4 / 100,000<br>▪ 1-4y: 75,4/100,00<br>▪ 15-19y: 37,2/100,00
				Clinical presentation	Proportion of patient with certain presentation	▪ 43% of Sg B cases presented purpura fulminans (vs 23% at national level)<br>▪ 49% of Sg B:14:P1.7,16 cases presented purpura fulminans
				Mortality	CRF	▪ 17% of Sg B cases died (vs 8,5% at a national level)<br>▪ 21% of Sg B:14:P1.7,16 cases died
Parent du Chatelet<br>J Infect<br>2017<br>[19]	Cohort study	5,772 IMD cases notified to the regional health agencies	2006-2015		Annual IMD incidence rate by year and by serogroup	▪ 2006: 1,23/100,000<br>▪ Sg B: 0,69/100,000<br>▪ Sg C: 0,29/100,000<br><br>▪ 2015: 0,73/100,000<br>▪ Sg B: 0,39/100,000<br>▪ Sg C: 0,19/100,000

Publication	Study design	Study population	Study date	Type of endpoint	Endpoint definition	Results and/or main findings
				IMD incidence		▪ The incidence rate of Sg W remained below 0.05 except in 2012 (0.07).<br>▪ Sg Y incidence rates remained below 0.05 before 2010 and varied between 0.05 and 0.09 between 2011 and 2015<br>▪ This decrease in annual IMD incidence rates was mainly related to the decrease in group B IMD
					Mean annual incidence rates by age group	▪ <1y: 11,11/100,000<br>▪ 1-4y: 3,45/100,000<br>▪ 5-14y: 0,89/100,000<br>▪ 15-24y: 1,84/100,000<br>▪ 25-59y: 0,35/100,000<br>▪ ≥60y: 0,45/100,000
					Distribution by serogroup	▪ Sg B: 60,9% (n=3,518/5,772)<br>▪ Sg C: 21,4% (n=1,236/5,772)<br>▪ Sg W: 3,8% (n=218/5,772)<br>▪ Sg Y: 5,8% (n=335/5,772)
					Mean annual incidence rates by serogroup and by age group	▪ SgB:<br>▪ <1y: 8,18/100,000<br>▪ 1-4y: 2,34/100,000<br>▪ 5-14y: 1,14/100,000<br>▪ 15-24y: 0,74/100,000<br>▪ 25-59y: 0,14/100,000<br>▪ ≥60y: 0,19/100,000<br>▪ Sg W:<br>▪ <1y: 0,28/100,000<br>▪ 1-4y: 0,09/100,000<br>▪ 5-14y: 0,02/100,000<br>▪ 15-24y: 0,05/100,000<br>▪ 25-59y: 0,02/100,000<br>▪ ≥60y: 0,04/100,000<br>▪ Sg C:<br>▪ <1y: 1,56/100,000<br>▪ 1-4y: 0,60/100,000<br>▪ 5-14y: 0,22/100,000<br>▪ 15-24y: 0,44/100,000<br>▪ 25-59y: 0,09/100,000<br>▪ ≥60y: 0,10/100,000<br>▪ Sg Y:<br>▪ <1y: 0,29/100,000<br>▪ 1-4y: 0,05/100,000<br>▪ 5-14y: 0,03/100,000<br>▪ 15-24y: 0,09/100,000<br>▪ 25-59y: 0,02/100,000<br>▪ ≥60y: 0,09/100,000<br>▪ Group C incidence decreased from 0.29 in 2006 to 0.13 in 2010 but increased thereafter in age groups not targeted by MCCV.

Publication	Study design	Study population	Study date	Type of endpoint	Endpoint definition	Results and/or main findings
					Strain genotype by serogroup	▪ Sg B: cc41/44 (28%) and cc32 (26%).<br>▪ Sg C: cc11 (92%)<br>▪ Sg W: cc11 (58%) and cc22 (25%).<br>▪ Sg Y: cc23 (63%)
				Clinical presentation	Proportion of patient with certain clinical presentation	▪ Bacteremia only: 12.2%<br>▪ Meningitis only: 43.0%<br>▪ Meningitis associated with bacteremia: 17.7%<br>▪ Purpura fulminans: 25.8%
					Distribution by serogroup of cases with Purpura fulminans	▪ Sg B: 25%<br>▪ Sg C: 29%<br>▪ Sg W: 11%<br>▪ Sg Y: 11%
				Long-term sequelae	Number of patients with sequelae	▪ N=321cases survived with sequelae diagnosed during the acute phase and before hospital discharge (5,7%)
				Mortality	CRF	▪ 10,4% of reported cases died
					Distribution by serogroup	▪ Sg B: 8,8%<br>▪ Sg C: 13,2%<br>▪ Sg W: 11,9%<br>▪ Sg Y: 15,5%
					Distribution by age	▪ <1y: 9,9%<br>▪ 1-4y: 8,9%<br>▪ 5-14y: 5,9%<br>▪ 15-24y: 10,3%<br>▪ 25-59y: 9,3%<br>▪ ≥60y: 20%
Perrocheau Euro Surveillance 2005 [44]	Cohort study	803 IMD reported cases	2003	IMD incidence	Incidence rate per person	▪ 1,8/100,000 inhabitants after adjusting for the under-reporting<br>▪ From 2002 to 2003, the number of cases increased by 18%. The increase could be explained by the new case definition.
					Distribution by serogroup	▪ Sg B: 59%<br>▪ Sg C: 32%<br>▪ Sg W135: 5%<br>▪ Sg Y: 3%

Publication	Study design	Study population	Study date	Type of endpoint	Endpoint definition	Results and/or main findings	
				Clinical presentation	Proportion of patient with certain clinical presentation	▪ Meningitis: 79%<br>▪ Septicemia: 13%<br>▪ Septicemia + meningitis: 18%<br>▪ Other type of IMD: 1,1%<br>▪ Purpura fulminans: 57%	
					Distribution by serogroup	<u>Patients with purpura fulminans</u><br>▪ Sg B: 50,3% (n=219/435)<br>▪ Sg C: 42,1% (n=183/435)<br>▪ Sg W135: 4,4% (n=19/435)<br>▪ Sg Y: 3,2% (n=14/435)	<u>Patient without purpura fulminans</u><br>▪ Sg B: 55,4% (n=580/1046)<br>▪ Sg C: 34,2% (n=358/1046)<br>▪ Sg W135: 6,3% (n=66/1046)<br>Sg Y: 4,0% (n=42/1046)
					Distribution by age	<u>Patients with purpura fulminans</u><br>▪ <2y: 24,5% (n=131/535)<br>▪ 2-14y: 35,3% (n=189/535)<br>▪ 15-24y: 23,9% (n=128/535)<br>▪ 25-99y: 16,3% (n=87/535)	<u>Patient without purpura fulminans</u><br>▪ <2y: 22,4% (n=263/1172)<br>▪ 2-14y: 28,7% (n=337/1172)<br>▪ 15-24y: 23,9% (n=280/1772)<br>▪ 25-99y: 24,9% (n=292/1772)
				Mortality	Distribution by year	▪ 2002: 16%<br>▪ 2003: 12%	
					Distribution by clinical presentation (2001-2003)	▪ 36,6% of patients with purpura fulminans died (vs 5,2% of cases without purpura fulminans)	
					Distribution by age	<u>Patients with purpura fulminans</u><br>▪ <2y: 42.7%<br>▪ 2-14y: 27.0%<br>▪ 15-24y: 24.2%<br>▪ 25-99y: 51.7%	<u>Patient without purpura fulminans</u><br>▪ <2y: 1.9%<br>▪ 2-14y: 2.4%<br>▪ 15-24y: 2.9%<br>▪ 25-99y: 12.0%
					Distribution by serogroup (2001-2003)	<u>Patients with purpura fulminans</u>	<u>Patient without purpura fulminans</u>

Publication	Study design	Study population	Study date	Type of endpoint	Endpoint definition	Results and/or main findings
						▪ Sg B: 33.8%<br>▪ Sg C: 37.2%<br>▪ Sg W135: 63.2%<br>▪ Other Sg: 35.7%<br>▪ Sg B: 2.8%<br>▪ Sg C: 8.1%<br>▪ Sg W135: 7.6%<br>▪ Other Sg: 9.5%
Pivette<br>BMC Public Health<br>2020<br>[22]	Case report	5 IMD reported cases in Brittany region	2016-2017	IMD incidence	Incidence rate of Sg B cc 162	▪ 5 cases belonged to B: P1.7–2, 4:F5–9:cc162 strain<br>▪ 6,4/100,000 (vs 0,3/100,000 at a national level excluding the department Côtes-d'Armor)
					Incidence of Sg B by age group	▪ 10-14y: 26,3/100,000 (0,3/100,000 at a national level excluding the department Côtes-d'Armor)<br>▪ 15-19y: 94,1/100,000 (0,6/100,000 at a national level excluding the department Côtes-d'Armor)
Rosain<br>Journal of Infectious Disease<br>2017<br>[31]	Cohort study	56 IMD cases with Terminal complement Pathway deficiencies (TCPD) admitted in Georges-Pompidou Hospital in Paris (aged between 14 months and 39 years)	1980-2015	IMD incidence	Number of cases by serogroup	▪ Sg B: 30% (n=18)<br>▪ Sg C: 3% (n=2)<br>▪ Sg W: 13% (n=8)<br>▪ Sg Y: 44% (n=27)<br>▪ The frequency of group Y in IMD was significantly higher in TPD patients compared with the general population.
					Number of cases by clonal complexes	▪ Hyperinvasive clonal complexes: 21% (n=11/53)<br>▪ CC23: 26% (n=14/53)<br>▪ Others: 53% (n=28/53).<br>▪ Thirteen of 14 strains from CC23 were from group Y isolates.
Sarlangue<br>Archive de pédiatrie<br>2006<br>[80]	Cohort study	1,703 children diagnosed with bacterial infection (including 782 cases of meningococcal meningitis)	2001-2004	IMD incidence	Proportion of N.m infections in patient with bacterial meningitis	▪ 45,9% (n=782)
					Number of cases by age	▪ < 2 m: 3,3% (n=26/782)<br>▪ 2-24 m: 39% (n=306/782)<br>▪ ≥ 24 m: 57% (n=450/782)
					Distribution by serogroup	▪ Sg B: 58,3% (n=456/782)<br>▪ Sg C: 32% (n=250/782)<br>▪ Others Sg: 13,5% (n=106/782)

Publication	Study design	Study population	Study date	Type of endpoint	Endpoint definition	Results and/or main findings
					Number of cases by age and serogroup	▪ Sg B<br>▪ < 2 m: 18<br>▪ 2-24 m: 177<br>▪ ≥ 24 m: 231<br>▪ Sg C<br>▪ < 2 m: 6<br>▪ 2-24 m: 88<br>▪ ≥ 24 m: 156<br>▪ Other Sg:<br>▪ < 2 m: 2<br>▪ 2-24 m: 41<br>▪ ≥ 24 m: 63<br><br>▪ After a steady increase in the incidence of invasive meningococcal infections (IMI) in France since 1998 and an acceleration of this phenomenon in 2003, partly due to the modification of the definition criteria in July 2002.<br>▪ A decrease in incidence was noted in 2004.<br>▪ The distribution by serogroup remaining unchanged.
Shen<br>Infectious Diseases and Therapeutics<br>2021<br>[48]	Cohort study	3,532 incidents cases of IMD, reported in the Medical Information System Program database (PMSI)	2012-2017	Long term sequelae	Number of patients with sequelae	▪ N= 823 patient had at least one sequela (23.3%)
				Mortality	CRF	▪ 12,9% of patients died following IMD<br>▪ 8.3% of reported cases died during hospitalization (n=293/3,532)<br>4,3% of reported cases died after the discharge (163/3,239)<br>42,3% of patient who died after the discharge had sequelae (n=69/163)
					Distribution by age	▪ 5,6% of patient aged <25y died during hospitalization (n=110/293)<br>▪ 5,8% of patient aged <25y died after discharge (n=115)

Publication	Study design	Study population	Study date	Type of endpoint	Endpoint definition	Results and/or main findings
Taha<br>J Clin Microbiol<br>2004<br>[29]	Cohort study	205 Isolates of N. meningitidis Sg W135	1994-2002	IMD incidence	Proportion of Sg W by year in the total number of invasive strains	▪ 1995: 1%<br>1996: 3,1%<br>1997: 1,6%<br>1998: 2,4%<br>1999: 3,9%<br>2000: 10,6%<br>2001: 7,6%<br>2002: 9,3%
					Proportion of isolates with Hajj strain-related phenotypes	▪ On average 55% of isolates were related to Hajj strain-related phenotypes<br>▪ According to year:<br>▪ 2000:69%<br>▪ 2001: 60%<br>2002: 52%<br>▪ Results suggest the continuous emergence of new genetic lineages of serogroup W135 independently of the 2000 global outbreak.
Taha<br>BMC Research Notes<br>2020<br>[28]	Cohort study	IMD confirmed cases	Jan-May 2018-2020	IMD incidence	Number of IMD cases by year	▪ Jan-May 2018: 202<br>▪ Jan-May 2019: 176<br>▪ Jan-May 2020: 129
					Number of IMD cases by serogroup and by year	▪ Sg B:<br>▪ 2018: 45,0% (n=91)<br>▪ 2019: 47,7% (n=84)<br>▪ 2020: 48,0% (n=62)<br>▪ Sg C:<br>▪ 2018: 24,2% (n=49)<br>▪ 2019: 14,8% (n=26)<br>▪ 2020: 8,5% (n=11)<br>▪ Sg W:<br>▪ 2018: 15,8% (n=32)<br>▪ 2019: 23,3% (n=41)<br>▪ 2020: 26,3% (n=34)<br>▪ Sg Y:<br>▪ 2018: 14,3% (n=29)<br>▪ 2019: 11,4% (n=20)<br>▪ 2020: 13,9% (n=18)

Publication	Study design	Study population	Study date	Type of endpoint	Endpoint definition	Results and/or main findings
						▪ The decrease during the lockdown, seemed to mainly involve IMD cases due to serogroups B and C and W but not IMD due to serogroup Y and other unusual serogroups or non-serogroupable isolates which did not decrease significantly, and which proportions increased during the lockdown 2020.
					Number of strains by year	Hyperinvasive clonal complexes: ▪ 2018: 123 ▪ 2019: 101 ▪ 2020: 63<br>Non hyperinvasive clonal complexes: ▪ 2018: 57 ▪ 2019: 65 ▪ 2020: 59<br>▪ The decrease during the lockdown involved mainly the highly transmissible and hyperinvasive isolates belonging to the clonal complex CC11 that almost disappeared.
Thabuis EuroCohort 2018 [21]	Case report	4 IMD cases (aged between 3-17y) with Sg B in the Auvergne Rhône Alpes region	2016	IMD incidence	Incidence rate	▪ 22,5/100,000 inhabitants (vs 0,3/100,000 in the rest of France)
					Strain genotype	▪ All Sg B cases belonged to B:P1.19,15:F4–28:cc32
				Clinical presentation	Proportion of patient with certain presentation	▪ Meningitis: 75% (n=3/4)<br>▪ Purpura fulminans: 25% (n=1/4)
				Long term sequelae	Proportion of patient with certain sequelae	▪ N=2 patient had at least one sequela
Weil-Olivier Infect Dis Ther 2021 [56]	Case-control study	3,532 incidents cases of IMD, reported in the Medical Information System Program database (PMSI)	2012-2017	Follow-up modalities	Follow-up modalities	▪ Cases were admitted to rehabilitation facilities and required home care<br>▪ Nearly two times the proportion of cases compared to controls consulted a hospital-based specialist as an outpatient.<br>▪ Cases also more frequently received nursing care, physiotherapy and speech therapy compared to controls.

Publication	Study design	Study population	Study date	Type of endpoint	Endpoint definition	Results and/or main findings
Weil-Olivier<br>Hum Vaccin Immunother<br>2022<br>[46]	Case-control study	3,532 incidents cases of IMD, reported in the Medical Information System Program database (PMSI)	2012-2017	IMD incidence	Distribution by age group	▪ <1y: 13,3%<br>▪ 1-4y: 13,2%<br>▪ 5-14y: 9,7%<br>▪ 15-19y: 11,0%<br>▪ 20-24y: 8,6%<br>▪ 25-59y: 25,1%<br>▪ ≥60y:19,2%
				Mortality	CRF	▪ 12,6% of patient died during the entire study follow-up
					Distribution by age group	▪ <1y: 5,1%<br>▪ 1-4y: 5,1%<br>▪ 5-14y: 4,1%<br>▪ 15-24y: 7,7%<br>▪ The mortality risk was highest for the 25–59-year age group, with a nearly five-fold elevation risk compared to controls
				Long term sequelae	Proportion of patient with certain sequelae	▪ 25,4% of patient has at least one sequela<br>▪ All individual sequelae were significantly more frequent (p < .0001) in cases than controls. Hearing/visual impairment and communication problems were conditions that presented the highest risk for cases compared to controls.<br>▪ In, general, frequency increased with age, and this was also the case for each individual sequela, with the exception of skin scarring and amputation
					Distribution by age	▪ Children <19y: 15,3% (n=255/1667)<br>▪ Adult >19y: 31,3% (n=490/1562)

Publication	Study design	Study population	Study date	Type of endpoint	Endpoint definition	Results and/or main findings
					Distribution by number of sequelae	▪ One sequela: 16,2% (n=525)<br>▪ Two sequelae: 5,8% (n=187)<br>▪ Three sequelae: 1,9% (n=60)<br>▪ Four sequelae: 1,0% (n=34)<br>▪ Five or more: 0,5% (n=17)
					Distribution by type of sequelae	▪ Epilepsy: 5,8%<br>▪ Anxiety: 5,5%<br>▪ Severe neurological disorder: 5,5%<br>▪ Motor deficits: 3,5%<br>▪ Depression: 2,5%<br>▪ Skin scarring: 2,3%<br>▪ Cochlear implant: 2,0%<br>▪ Bilateral hearing loss: 0,8%<br>▪ Unilateral hearing loss: 2,0%<br>▪ Speech or communication problems: 1,7%<br>▪ Blindness/severe visual impairment: 1,7%<br>▪ Amputation: 1,5%<br>▪ Renal disease: 1,5%<br><br>▪ The most frequently documented sequelae were epilepsy (N = 205; 5.8%), anxiety (N = 196; 5.5%), and severe neurological disorders (N = 193; 5.5%).
				Hospitalization details	Hospitalization details	▪ Mean length of hospitalization: 14.8 days (median: 8 days; IQR: 6-14 days)<br>▪ The stay duration was highly dependent on age, with cases aged ≥60 years being hospitalized for a mean duration of 25 days<br>▪ ICU admission: 44,6%<br>▪ Discharge status:<br>▪ Rehabilitation center: 2,9%<br>▪ Home: 84,2%
Zarantonelli<br>Clin Microbiol Infect<br>2008<br>[26]	Cohort study	546 isolates from IMD cases	2000-2004	IMD incidence	Distribution of isolates by serogroup	▪ Sg B: 56%<br>▪ Sg C: 30%<br>▪ Sg W135: 9%<br>▪ Sg Y: 3%

Publication	Study design	Study population	Study date	Type of endpoint	Endpoint definition	Results and/or main findings
					Distribution by clonal complex (most frequent ones)	▪ ST-11: 33,5% (n=183)<br>▪ ST 41/44: 26,2% (n=143)<br>▪ ST 32: 10% (n=55)<br>▪ ST 8: 8,4% (n=46)<br>▪ ST 269: 5,7% (n=31)<br>▪ Clonal complex ST-11, which was mostly serogroup C, also harboured a significantly high proportion of serogroups B and W135 (28.4%, p <0.00001).
				Mortality	CRF	▪ Immediate fatality was significantly higher for cases involving clonal complex ST-11, which had a case fatality rate of 16%, compared to 7.1% for the entire population of isolates

Table S5. Characteristics of national studies including IMD incidence data distributed by age and serogroup

Publication	Time of publication	Study population	Time frame	Number of cases	Incidence rate*	Age distribution	Serogroup distribution
Taha<br>J Clin Microbiol<br>[29]	2004	Strain isolates of N. meningitidis from invasive infections caused by Sg W	1994-2002	205	NR	NR	Sg W:<br>1995: 1%<br>1996: 3,1%<br>1997: 1,6%<br>1998: 2,4%<br>1999: 3,9%<br>2000: 10,6%<br>2001: 7,6%<br>2002: 9,3%
Antignac<br>Clinical Infect Dis<br>[72]	2003	Clinical isolates of N. meningitidis from invasive infections	1999-2002	2,167	<1/100,000	NR	Sg B: 58%<br>Sg C: 29%<br>Sg W: 8%<br>Sg Y: NR
Deghmane<br>The journal of infectious diseases<br>[18]	2010	Strain isolates of N. meningitidis from invasive infections	1999-2008	6,528	1999: 0,98/100,000<br>2002: 1,43/100,000<br>2003: 1,61/100,000<br>2008: 1,2/100,000<br>(After correcting for under-reporting)	NR	Sg B: 62%<br>Sg C: 29%<br>SgW135: 5%<br>Sg Y: 3%
Aguilera<br>Emerging Infectious Diseases<br>[52]	2002	Hajj Piligrims and non piligrims diagnosed with IMD caused by Sg W135	2000	24	21/100,000	<1 year: 13%<br>1–4: 29%<br>5–9: 8%<br>10–19: 8%<br>20–49: 13%<br>50–65: 17%<br>>65: 13%	NA
Zarantonelli<br>Clin Microbiol Infect<br>[26]	2008	Strain isolates of N. meningitidis from invasive infections	2000-2004	546	NR	NR	Sg B: 56%<br>Sg C: 30%<br>Sg W135: 9%<br>Sg Y: 3%
Contou<br>Intensive Care Med<br>[47]	2018	Adults (aged >18y) diagnosed with purpura fulminans caused by N.m	2000-2016	195	NR	NR	Sg B: 39%<br>Sg C: 34%
Hong<br>J Infect<br>[30]	2018	Strain isolates of N. meningitidis from invasive infections caused by Sg W	2000-2016	527	NR	2015-2016<br>0-14y: 19,5%<br>15-24y: 27,3%<br>25-59: 27,3%<br>>60y: 26%	Sg W: 100%
Cohen<br>Archives de Pédiatrie<br>[75]	2003	Children (aged <18y) diagnosed with meningococcal meningitis	2001	194	NR	< 1 month: 4,4%<br>1-3m: 24,2%<br>3-24 m: 41,6%<br>> 24 m: 56,7%	NR
Bingen<br>Clinical Infectious Diseases<br>[51]	2005	Children (aged <18y) diagnosed with meningococcal meningitis	2001-2003	599	NR	1-2m: 2%<br>2-12m: 24,9%<br>1-2y: 15,3%<br>2-15y: 53,7%	Sg B: 58,3%<br>Sg C: 38,1%<br>Sg Other: 16,2%

Publication	Time of publication	Study population	Time frame	Number of cases	Incidence rate*	Age distribution	Serogroup distribution
						15-18y: 4%	
Sarlangue<br>Archive de pédiatrie<br>[80]	2006	Children (aged <18y) diagnosed with meningococcal meningitis	2001-2004	782	NR	< 2 m: 3,3%<br>2-24 m: 39%<br>≥ 24 m: 57%	Sg B: 58,3%<br>Sg C: 32%<br>Other Sg: 13,5%
Levy<br>Pediat Infect Dis<br>[38]	2010	Children (aged <18y) diagnosed with meningococcal meningitis	2001-2005	962	NR	1d-<1y: 27,8%<br>≥1y->5y: 39,9%<br>≥5-<10y: 18,9%<br>≥10-18y: 13,4%	Sg B: 62,3%<br>Sg C: 33,7%<br>Sg W135: 2,9%<br>Sg Y: 0,6%
Levy<br>Archives de Pédiatrie (a)<br>[39]	2008	Children (aged <18y) diagnosed with meningococcal meningitis	2001-2007	1,344	NR	NR	Sg B: 59%<br>Sg C: 29%<br>Sg W: 2,4%<br>Sg Y: 0,7%
Levy<br>Archives de Pédiatrie (b)<br>[45]	2008	Children (aged <18y) diagnosed with meningococcal meningitis	2001-2007	1,344	NR	< 1m: 1,0%<br>1-2m: 2,0%<br>2-12m: 26,9%<br>12-24m: 13,7%<br>24m-5y: 24,8%<br>> 5y: 31,5%	Sg B: 59%<br>Sg C: 29%
Gaschignard<br>Pediatr Infect Dis J<br>[34]	2013	Children (aged <17y) diagnosed with IMD caused by Sg W	2001-2008	119	NR	The highest number of cases was in infants between 6 and 9 months old.	Sg W: 100%
Levy<br>Achives de Pédiatrie<br>[49]	2012	Children (aged <18y) diagnosed with meningococcal meningitis	2001-2009	1,661	NR	NR	Sg B: 61,3%<br>Sg C: 27,0%<br>Sg W135: 2,4%<br>Sg Y: 0,6%
Levy<br>Achives de Pédiatrie<br>[50]	2014	Children (aged <18y) diagnosed with meningococcal meningitis	2001-2012	1,991	NR	< 1m: 0,9%<br>1-2m: 2,4%<br>2-12m: 27,7%<br>12-24m: 14,6%<br>24m-5y: 23,4%<br>> 5y: 31,0%	Sg B: 64%<br>Sg C: 24%
Bilal<br>Pediatric Infectious Disease<br>[37]	2016	Neonatal cases (<28 days of age) diagnosed with meningococcal meningitis	2001-2013	23	NR	NR	Sg B: 78%<br>Sg C: 13%<br>Sg others: 9%
Perrocheau<br>Euro Surveillance<br>[44]	2005	IMD reported cases	2003	803	1,3/100,00<br>1,8/ 100,000 (after correcting for under-reporting)	NR	Sg B: 59%<br>Sg C: 32%<br>Sg W135: 5%<br>Sg Y: 3%
Parent Du châtelet<br>J Infect<br>[19]	2017	IMD cases notified to regional health agencies	2006-2015	5,772	2006: 1,23/100,000<br><br>2015: 0,73/100,000	<1y: 11,11/100,000<br>1-4y: 3,45/100,000<br>5-14y: 0,89/100,000<br>15-24y: 1,84/100,000<br>25-59y: 0,35/100,000<br>≥60y: 0,45/100,000	Sg B: 60,9%<br>Sg C: 21,4%<br>Sg W: 3,8%<br>Sg Y: 5,8%

Publication	Time of publication	Study population	Time frame	Number of cases	Incidence rate*	Age distribution	Serogroup distribution
Weil-Olivier<br>Hum Vaccin Immunother<br>[46]	2022	Incidents cases of IMD, reported in the Medical Information System Program database (PMSI)	2012-2017	3,532	NR	<1y: 13,3%<br>1-4y: 13,2%<br>5-14y: 9,7%<br>15-19y: 11,0%<br>20-24y: 8,6%<br>25-59y: 25,1%<br>≥60y:19,2%	NA
Hong<br>Human Vaccines and Immunotherapeutics<br>[78]	2021	IMD cases biologically confirmed	2013-2014<br>2018-2019	428<br>366	NR	NR	Sg B: 55%<br>Sg B: 51,4%
Duval<br>Advances in Therapy<br>[40]	2022	Adults (aged >18y) diagnosed with meningococcal meningitis	2013-2015	111	NR	NR	Sg B: 52%<br>Sg C: 39%<br>Sg Y: 9%
Deghmane<br>J Infect<br>[42]	2020	Strain isolates of N. meningitidis from invasive infections caused by ST-9316	2013-2018	27	Sg W:<br>2013: 0,04/100,000<br>2018: 0,09/100,000	NR	Sg B: 22%<br>Sg C: 4%<br>Sg W: 74%
Huang<br>Plos One<br>[43]	2022	IMD cases notified in PMSI data base and reported in the Institut de Veille Sanitaire (InVS)	2014-2016	1,344	NR	<1y: 15%<br>1-4y: 14%<br>5-14y: 11%<br>15-24y: 13%<br>25-59y: 17%<br>≥60y: 14%	NR
Taha<br>BMC Research Notes<br>[28]	2020	IMD cases biologically confirmed	Jan-march<br>2018-2020	2018: 202<br>2019: 176<br>2020: 129	NR	NR	Sg B:<br>2018: 45,0%<br>2019: 47,7%<br>2020: 48,0%<br>Sg C:<br>2018: 24,2%<br>2019: 14,8%<br>2020: 8,5%<br>Sg W:<br>2018: 15,8%<br>2019: 23,3%<br>2020: 26,3%<br>Sg Y:<br>2018: 14,3%<br>2019: 11,4%<br>2020: 13,9%
Deghmane<br>Microorganisms<br>[20]	2022	IMD cases biologically confirmed	2017-2021	2017: 474<br>2018: 397<br>2019: 416<br>2020: 202<br>2021: 106	NR	NR	Sg B: 49,6%<br>Sg C: 17,9%<br>SgW135: 17%<br>Sg Y: 13,9%

d: day; NA: Non applicable; NR: Non reported; m: month; Sg: serogroup; y: year
*Incidence rate is defined as the total number of IMD cases per 100,000 individuals exposed to the risk of IMD.

Table S6. Characteristics of regional studies including IMD incidence data distributed by age and serogroup

Reference	Time of publication	State/Region	Study population	Time frame	Number of cases	Incidence rate*	Age distribution	Serogroup distribution and/or main strain genotype
Rosain Journal of Infectious Disease [31]	2017	Paris	IMD cases with Terminal complement Pathway deficiencies (TCPD)	1980-2015	56	NR	NR	Sg B: 30%<br>Sg C: 3%<br>Sg W: 13%<br>Sg Y: 44%<br><br>CC23: 26% of all strains<br>93% of CC23 were from Sg Y
Caron Lancet Infectious Disease (a) [24]	2011	Seine-Maritime	IMD reported cases caused by Sg B	1990-2001	118	NR	NR	Sg B: 85%<br>Sg B:14:P1.7,16: 34%
Faye Archives de Pédiatrie [32]	2005	Paris	IMD reported cases caused by Sg W	2000-2002	5	NR	NR	Sg W ET-37/ ST11
Grodet Clinical Microbiology and Infection [77]	2004	Indre-et-Loire	IMD reported cases caused by Sg B	2000-2002	8	NR	14-28y	B:15:P1.12
Lorton Pediatr Crit Care Med (a) [36]	2020	Loire Atlantique et Vendée	Children older than 3 months (and <18y) diagnosed with meningococcal infections	2000-2006	54	2,7/ 100 000	NR	Sg B: 29%<br>Sg C: 14%
Lévy-Bruhl Eurosurveillance [79]	2002	Puy-de-Dôme	IMD reported cases	2001-2002	17	Sg C:<br>1,7 /100 000<br>(vs 0,3/100 000 at national level)	<5y: 55%	Sg B: 13%<br>Sg C: 73%
Dubos Arch Pediat [33]	2009	Nord Pas de Calais	Incidents cases of IMD, reported in the Medical Information System Program database (PMSI) and at InVS	2002-2005	319	2002: 6,9/100,000<br>2003: 8,3/100,000<br>2004: 6,9/100,000<br>2005: 9,4/100,000	2005 :<br><1y: 33,4/100,000<br>1-4y: 8,3/100,000<br>5-17y: 1,4/100,000	Sg B: 56%<br>Sg C: 16%<br>Sg A,Y ou W135: 3%
Parent du Chatelet Archiv Pediat [25]	2007	Dieppe	IMD reported cases caused by Sg B	2003-2006	NR	11,5 / 100,000<br>(vs 1,2/100,000 at a national level)	<1y: 46,4 / 100,000<br>1-4y: 75,4/100,00<br>15-19y: 37,2/100,00	B:14:P1.7,16
Caron Lancet Infectious Disease (b) [24]	2011	Seine-Maritime	IMD reported cases caused by Sg B	2003-2006	91	11,4/100,000 in Dieppe area<br><br>1,2/100,00 in Seine maritime	NR	Sg B: 80%<br>Sg B:14:P1.7,16 : 66%

Reference	Time of publication	State/Region	Study population	Time frame	Number of cases	Incidence rate*	Age distribution	Serogroup distribution and/or main strain genotype
Delisle<br>Euro Surveillance<br>[23]	2010	Landes-Aquitaine	IMD reported cases caused by Sg B	2008-2009	11	3,0/100,000 (vs 0,6/100,000 at national level)	<5y: 27% (vs 46% in the rest of France)<br>5-14y: 9% (vs 10% in the rest of France)<br>15-19y: 18% (vs 15% in the rest of France)<br>20-24y: 36% (vs 10% in the rest of France)<br>≥25y: 9% (vs 19% in the rest of France)	Sg B/ST-269
Lorton<br>Pediatr Crit Care Med (b) [36]	2020	Loire Atlantique et Vendée	Children older than 3 months (and <18y) diagnosed with meningococcal infections	2009-2014	71	0,8/ 100 000	NR	Sg B: 44%<br>Sg C: 14%
Lorton<br>Pediatric and perinatal epidemiology<br>[53]	2018	Loire Atlantique et Vendée	Children (aged <18y) diagnosed with meningococcal infections	2009-2014	75	NR	NR	Sg B: 63%<br>Sg C: 20%<br>Sg W: 1,3%<br>Sg Y: 1,3%
Arlet<br>Presse Médicale<br>[54]	2010	Paris	Patient aged 58y admitted for endophtalmis and arthritis secondary to meningococcal meningitis caused by Sg C	2010	1	NR	NR	Sg C
Aubert<br>Eurosurveillance<br>[73]	2015	Paris	Strain isolates of N. meningitidis from invasive infections caused by Sg C	2013-2014	56	0,05/100,000 (vs 0,02/100,000 at national level)	25 to 59y: 47%<br>≥60 y: 14%	C:P1.5-1,10-8:F3-6:cc11
Deghmane<br>J Infect<br>[42]	2020	Hauts-de-France	Strain isolates of N. meningitidis from invasive infections caused by Sg W	2013-2014	29	2013 : 0,05/100,000<br><br>2018 : 0,12/100,00	Sg W ST-9316<br>< 5y : 67% (vs 9% in patient with IMD W/CC11)<br><br>6-14 y: 0% (0% in patient with IMD W/CC11)<br><br>15-24 y: 0% (vs 18% in patient with IMD W/CC11)<br><br>25-49y: 7% (vs 9% in patient with IMD W/CC11)<br><br>≥ 50 y: 27% (vs 55% in patient with IMD W/CC11)	Sg W/ST-9316: 52%
Barret<br>Médecine et Maladie infectieuse<br>[74]	2020	Dijon	IMD cases caused by Sg W	2016	3	10,8/100,000	NR	W:P1.5,2:F1-1:cc11(UK-2013 strain)

Reference	Time of publication	State/Region	Study population	Time frame	Number of cases	Incidence rate*	Age distribution	Serogroup distribution and/or main strain genotype
Thabuis Eurosurveillance [21]	2018	Auvergne Rhône Alpes	IMD reported cases caused by Sg B	2016	4	22,5/100,000 (vs 0,3/100,000 in the rest of France)	NR	B:P1.19,15:F4– 28:cc32
Bassi Eurosurveillance [41]	2017	Paris	IMD cases caused by Sg W	2017	2	NR	NR	W:P1.5,2:F1–1:cc11
Pivette BMC Public Health [22]	2020	Brittany	IMD reported cases caused by Sg B	2016-2017	5	6,4/100,000 (vs 0,3/100,000 at a national level excluding the department Côtes-d'Armor)	10-14y: 26,3/100,000 (0,3/100,000 at a national level excluding the department Côtes-d'Armor)<br><br>15-19y: 94,1/100,000 (0,6/100,000 at a national level excluding the department Côtes-d'Armor)	B: P1.7–2, 4:F5–9:cc162

d: day; NA: Non applicable; NR: Non reported; m: month; Sg: serogroup; y: year
*Incidence rate is defined as the total number of IMD cases per 100,000 individuals exposed to the risk of IMD.

Table S7. Characteristics of included studies assessing and describing the type and the proportion of sequelae in France

	**Duval and al**<br>**Advances in Therapy**<br>**2022**<br>**[40]**	**Huang and al**<br>**Plos One**<br>**2022**<br>**[43]**	**Weil-Olivier**<br>**Human vaccines and immunotherapeutics**<br>**2022**<br>**[46]**
Clinical presentation	Meningococcal meningitis	Invasive meningococcal diseases	Invasive meningococcal diseases
Year of follow-up	2013-2015	2014-2016	2012-2017
Study design	National prospective multicenter cohort	Observational cohort study	Observational case–control study
Population	Adults aged ≥ 18y	All age	All age
N=	71	1344	3532
Mean age at the time of the diagnosis	30y (21,4y-56,0y)	26.5 y (median: 19y)	29.7 ± 27.6 y (median: 21 years [IQR: 4–52])
Mean period of follow-up	12 m	17,1 m	2.8 ± 1.9 y (median: 2.8 years [IQR: 0–6.0 years])
Sequelae	Nb of survivors =71<br>None = 91,3%<br>At least one = **5,6%**	Nb of survivors = 1264<br>None = 1003<br>At least one = 261 **(19,4%)**	N= 3239<br>None = 2,416 (74.6%)<br>At least one = 564 **(25,4%)**
Physical	NR	Amputation or skin necrosis or skin grafting: 2.7% (n = 36)<br>Chronic renal failure: 1,9% (n=25)<br>Arthritis: 1,4% (n=19)	Skin scarring: 2.3% (n=81)<br>Amputation: 1.5% (n=51)<br>Renal disease: 1.3% (n=45)
Neurological	Persistent headache: 32.9% (n=23)	Neurological sequelae: 11.5% (n = 154)	Motor deficits: 3.5% (n=123)<br>Epilepsy: 5.8% (n=205)<br>Severe neurological disorder: 5.5% (n=193)<br>Speech or communication problems: 1.7% (n=61)
Sensorial	Hearing impairment: 15.5% (n=11)	Auditive impairment: 2.8% (n=37)	Cochlear implant: 2.0% (n=70)<br>Bilateral hearing loss: 0.8% (n=30)<br>Unilateral hearing loss: 2.0% (n=69)<br>Severe visual impairment/blindness: 1.7% (n=60)
Cognitive	Intellectual disability: 10.0% (n=7)	Cognitive impairment: 1.7% (n=23)	NR

Psychological	Depressive symptoms: 34.3% (n=24)<br>Sleep disorders: 42.9% (n=30)	NR	Depression: 2.5% (n=87)<br>Anxiety: 5.5% (n=196)
Quality of life score	MCS: 50.8 [39.6–55.5]<br>PCS: 53.4 [44.3–55.5]<br><br>48,6% of patients had PCS score lower than the 25th percentile of the score distribution in the French general population<br><br>28,6% of patients had MCS score lower than the 25th percentile of the score distribution in the French general population	NR	NR

d: day; N: Number of patients included; NR: Non reported; m: month; y: year
Children: patient aged < 18 years old; Adult: patient aged > 18 years old; all: both children and adult
Regional data or data from case report studies were excluded.

References

18. Deghmane, A.E.; Parent du Chatelet, I.; Szatanik, M.; Hong, E.; Ruckly, C.; Giorgini, D.; Levy-Bruhl, D.; Alonso, J.M.; Taha, M.K. Emergence of new virulent Neisseria meningitidis serogroup C sequence type 11 isolates in France. J. Infect. Dis. 2010, 202, 247–250. https://doi.org/10.1086/653583.

19. Parent du Chatelet, I.; Deghmane, A.E.; Antona, D.; Hong, E.; Fonteneau, L.; Taha, M.K.; Levy-Bruhl, D. Characteristics and changes in invasive meningococcal disease epidemiology in France, 2006-2015. J. Infect. 2017, 74, 564–574. https://doi.org/10.1016/j.jinf.2017.02.011.

20. Deghmane, A.E.; Taha, M.K. Changes in Invasive Neisseria meningitidis and Haemophilus influenzae Infections in France during the COVID-19 Pandemic. Microorganisms 2022, 10, 907. https://doi.org/10.3390/microorganisms10050907.

21. Thabuis, A.; Tararbit, K.; Taha, M.K.; Dejour-Salamanca, D.; Ronin, V.; Parent du Chatelet, I.; Spaccaferri, G. Community outbreak of serogroup B invasive meningococcal disease in Beaujolais, France, February to June 2016: From alert to targeted vaccination. Eurosurveillance 2018, 23, 1700590. https://doi.org/10.2807/1560-7917.ES.2018.23.28.1700590.

22. Pivette, M.; Taha, M.K.; Barret, A.S.; Polard, E.; Hautier, M.B.; Dufour, J.B.; Faisant, M.; King, L.A.; Antona, D.; Levy-Bruhl, D.; et al. Targeted vaccination campaigns of teenagers after two clusters of B invasive meningococcal disease in Brittany, France, 2017. BMC Public Health 2020, 20, 1382. https://doi.org/10.1186/s12889-020-09487-7.

23. Delisle, E.; Larrieu, S.; Simoes, J.; Laylle, N.; De Pommerol, M.; Taha, M.K.; Termignon, J.L.; Parent du Chatelet, I. Community outbreak of group B meningococcal disease in southwest France--December 2008 to September 2009. Eurosurveillance 2010, 15, 19665.

24. Caron, F.; du Chatelet, I.P.; Leroy, J.P.; Ruckly, C.; Blanchard, M.; Bohic, N.; Massy, N.; Morer, I.; Floret, D.; Delbos, V.; et al. From tailor-made to ready-to-wear meningococcal B vaccines: Longitudinal study of a clonal meningococcal B outbreak. Lancet Infect. Dis. 2011, 11, 455–463. https://doi.org/10.1016/S1473-3099(11)70027-5.

25. Parent du Chatelet, I.; Taha, M.K.; Sesboue, C.; Rouaud, P.; Perrocheau, A.; Levy-Bruhl, D. [Increased incidence of invasive meningococcal disease in Seine-Maritime. The evolving epidemiology due to the B:14:P1.7,16 strain]. Arch. Pediatr. 2007, 14, 537–540. https://doi.org/10.1016/j.arcped.2007.02.038.

26. Zarantonelli, M.L.; Lancellotti, M.; Deghmane, A.E.; Giorgini, D.; Hong, E.; Ruckly, C.; Alonso, J.M.; Taha, M.K. Hyperinvasive genotypes of Neisseria meningitidis in France. Clin. Microbiol. Infect. 2008, 14, 467–472. https://doi.org/10.1111/j.1469-0691.2008.01955.x.

27. Levy-Bruhl, D.; Fonteneau, L.; Vaux, S.; Barret, A.S.; Antona, D.; Bonmarin, I.; Che, D.; Quelet, S.; Coignard, B. Assessment of the impact of the extension of vaccination mandates on vaccine coverage after 1 year, France, 2019. Eurosurveillance 2019, 24, 1900301. https://doi.org/10.2807/1560-7917.ES.2019.24.26.1900301.

28. Taha, M.K.; Deghmane, A.E. Impact of COVID-19 pandemic and the lockdown on invasive meningococcal disease. BMC Res. Notes 2020, 13, 399. https://doi.org/10.1186/s13104-020-05241-9.

29. Taha, M.K.; Giorgini, D.; Ducos-Galand, M.; Alonso, J.M. Continuing diversification of Neisseria meningitidis W135 as a primary cause of meningococcal disease after emergence of the serogroup in 2000. J. Clin. Microbiol. 2004, 42, 4158–4163. https://doi.org/10.1128/JCM.42.9.4158-4163.2004.

30. Hong, E.; Barret, A.S.; Terrade, A.; Denizon, M.; Antona, D.; Aouiti-Trabelsi, M.; Deghmane, A.E.; Parent du Chatelet, I.; Levy-Bruhl, D.; Taha, M.K. Clonal replacement and expansion among invasive meningococcal isolates of serogroup W in France. J. Infect. 2018, 76, 149–158. https://doi.org/10.1016/j.jinf.2017.10.015.

31. Rosain, J.; Hong, E.; Fieschi, C.; Martins, P.V.; El Sissy, C.; Deghmane, A.E.; Ouachee, M.; Thomas, C.; Launay, D.; de Pontual, L.; et al. Strains Responsible for Invasive Meningococcal Disease in Patients With Terminal Complement Pathway Deficiencies. J. Infect. Dis. 2017, 215, 1331–1338. https://doi.org/10.1093/infdis/jix143.

32. Faye, A.; Mariani-Kurkjian, P.; Taha, M.K.; Louzeau, C.; Bingen, E.; Bourrillon, A. [Clinical aspects and outcome of meningococcal disease due to Neisseria meningitidis of serogroup W135 in 5 children]. Arch. Pediatr. 2005, 12, 291–294. https://doi.org/10.1016/j.arcped.2004.11.018.

33. Dubos, F.; Marechal, I.; Tilmont, B.; Courouble, C.; Leclerc, F.; Martinot, A. [Incidence of invasive meningococcal diseases in children in Northern France: Usefulness and limits of the discharge code database for correcting compulsory notification data]. Arch. Pediatr. 2009, 16, 984–990. https://doi.org/10.1016/j.arcped.2009.03.006.

34. Gaschignard, J.; Levy, C.; Deghmane, A.E.; Dubos, F.; Muszlak, M.; Cohen, R.; Bingen, E.; Faye, A.; Taha, M.K. Invasive serogroup w meningococcal disease in children: A national survey from 2001 to 2008 in France. Pediatr. Infect. Dis. J. 2013, 32, 798–800. https://doi.org/10.1097/INF.0b013e31828e9e91.

35. Floret, D. Pediatric deaths due to community-acquired bacterial infections. A French survey in Pediatric Intensive Care Units. Arch. Pédiatrie 2001, 8, 705–711.

36. Lorton, F.; Chalumeau, M.; Martinot, A.; Assathiany, R.; Roue, J.M.; Bourgoin, P.; Chantreuil, J.; Boussicault, G.; Gaillot, T.; Saulnier, J.P.; et al. Epidemiology of Community-Onset Severe Bacterial Infections in Children and Its Evolution: A Population-Based Study in France. Pediatr. Crit Care Med. 2020, 21, e325–e332. https://doi.org/10.1097/PCC.0000000000002300.

37. Bilal, A.; Taha, M.K.; Caeymaex, L.; Cohen, R.; Levy, C.; Durrmeyer, X.; Groupe des Pediatres et microbiologistes de l'Observatoire National des, M.; Members of the National Reference Center for, M. Neonatal Meningococcal Meningitis In France From 2001 To 2013. Pediatr. Infect. Dis. J. 2016, 35, 1270–1272. https://doi.org/10.1097/INF.0000000000001296.

38. Levy, C.; Taha, M.K.; Weil Olivier, C.; Quinet, B.; Lecuyer, A.; Alonso, J.M.; Aujard, Y.; Bingen, E.; Cohen, R.; Groupe des pédiatres et microbiologistes de l'Observatoire National des Méningites. Association of meningococcal phenotypes and genotypes with clinical characteristics and mortality of meningitis in children. Pediatr. Infect. Dis. J. 2010, 29, 618–623. https://doi.org/10.1097/INF.0b013e3181d3ce32.

39. Levy, C.; Bingen, E.; Aujard, Y.; Boucherat, M.; Floret, D.; Gendrel, D.; Cohen, R.; Groupe des pediatres et microbiologistes de l'Observatoire National des, M. [Surveillance network of bacterial meningitis in children, 7 years of survey in France]. Arch Pediatr. 2008, 15, (Suppl. 3), S99-S104. https://doi.org/10.1016/S0929-693X(08)75491-5.

40. Duval, X.; Taha, M.K.; Lamaury, I.; Escaut, L.; Gueit, I.; Manchon, P.; Tubiana, S.; Hoen, B.; group, C.s. One-Year Sequelae and Quality of Life in Adults with Meningococcal Meningitis: Lessons from the COMBAT Multicentre Prospective Study. Adv. Ther. 2022, 39, 3031–3041. https://doi.org/10.1007/s12325-022-02149-7.

41. Bassi, C.; Taha, M.K.; Merle, C.; Hong, E.; Levy-Bruhl, D.; Barret, A.S.; Mounchetrou Njoya, I. A cluster of invasive meningococcal disease (IMD) caused by Neisseria meningitidis serogroup W among university students, France, February to May 2017. Eurosurveillance 2017, 22, 30574. https://doi.org/10.2807/1560-7917.ES.2017.22.28.30574.

42. Deghmane, A.E.; Haeghebaert, S.; Hong, E.; Jousset, A.; Barret, A.S.; Taha, M.K. Emergence of new genetic lineage, ST-9316, of Neisseria meningitidis group W in Hauts-de-France region, France 2013-2018. J. Infect. 2020, 80, 519–526. https://doi.org/10.1016/j.jinf.2020.01.020.

43. Huang, L.; Fievez, S.; Goguillot, M.; Marie, L.; Benard, S.; Elkaim, A.; Tin Tin Htar, M. A database study of clinical and economic burden of invasive meningococcal disease in France. PLoS ONE 2022, 17, e0267786. https://doi.org/10.1371/journal.pone.0267786.

44. Perrocheau, A.; Taha, M.K.; Levy-Bruhl, D. Epidemiology of invasive meningococcal disease in France in 2003. Eurosurveillance 2005, 10, 238–241.

45. Levy, C.; Taha, M.K.; Weill Olivier, C.; Quinet, B.; Lecuyer, A.; Alonso, J.M.; Cohen, R.; Bingen, E.; Groupe des pédiatres et microbiologistes de l'Observatoire National des Méningites. Characteristics of meningococcal meningitis in children in France. Arch. Pediatr. 2008, 15, (Suppl. 3), S105-110. https://doi.org/10.1016/S0929-693X(08)75492-7.

46. Weil-Olivier, C.; Taha, M.K.; Bouee, S.; Emery, C.; Loncle-Provot, V.; Nachbaur, G.; Beck, E.; Pribil, C. Care pathways in invasive meningococcal disease: A retrospective analysis of the French national public health insurance database. Hum. Vaccin. Immunother. 2022, 18, 2021764. https://doi.org/10.1080/21645515.2021.2021764.

47. Contou, D.; Sonneville, R.; Canoui-Poitrine, F.; Colin, G.; Coudroy, R.; Pene, F.; Tadie, J.M.; Cour, M.; Beduneau, G.; Marchalot, A.; et al. Clinical spectrum and short-term outcome of adult patients with purpura fulminans: A French multicenter retrospective cohort study. Intensive Care Med. 2018, 44, 1502–1511. https://doi.org/10.1007/s00134-018-5341-3.

48. Shen, J.; Bouee, S.; Aris, E.; Emery, C.; Beck, E.C. Long-Term Mortality and State Financial Support in Invasive Meningococcal Disease-Real-World Data Analysis Using the French National Claims Database (SNIIRAM). Infect. Dis. Ther. 2021, 11, 249–262. https://doi.org/10.1007/s40121-021-00546-z.

49. Levy, C.; Taha, M.K.; Bingen, E.; Cohen, R. Méningites à méningocoques de l’enfant en France: Résultats de l’observatoire ACTIV/GPIP. Arch. Pédiatrie 2012, 19, S49–S54. https://doi.org/10.1016/s0929-693x(12)71273-3.

50. Levy, C.; Varon, E.; Taha, M.K.; Béchet, S.; Bonacorsi, S.; Cohen, R.; Bingen, E. Change in French bacterial meningitis in children resulting from vaccination. Arch. Pédiatrie 2014, 21, 736-744. https://doi.org/.

51. Bingen, E.; Levy, C.; de la Rocque, F.; Boucherat, M.; Varon, E.; Alonso, J.M.; Dabernat, H.; Reinert, P.; Aujard, Y.; Cohen, R.; et al. Bacterial meningitis in children: A French prospective study. Clin. Infect. Dis. 2005, 41, 1059–1063. https://doi.org/10.1086/432944.

52. Aguilera, J.F.; Perrocheau, A.; Meffre, C.; Hahne, S.; Group, W.W. Outbreak of serogroup W135 meningococcal disease after the Hajj pilgrimage, Europe, 2000. Emerg. Infect. Dis. 2002, 8, 761–767. https://doi.org/10.3201/eid0808.010422.

53. Lorton, F.; Chalumeau, M.; Assathiany, R.; Martinot, A.; Bucchia, M.; Roue, J.M.; Bourgoin, P.; Chantreuil, J.; Boussicault, G.; Gaillot, T.; et al. Vaccine-preventable severe morbidity and mortality caused by meningococcus and pneumococcus: A population-based study in France. Paediatr. Perinat. Epidemiol. 2018, 32, 442–447. https://doi.org/10.1111/ppe.12500.

54. Arlet, J.B.; de Lajudie, E.; Despujol, C.; Ranque, B.; Pouchot, J. Meningitis and loss of visual acuity. Presse. Med. 2010, 39, 617–619. https://doi.org/10.1016/j.lpm.2010.02.033.

55. Briand, C.; Levy, C.; Baumie, F.; Joao, L.; Bechet, S.; Carbonnelle, E.; Grimprel, E.; Cohen, R.; Gaudelus, J.; de Pontual, L. Outcomes of bacterial meningitis in children. Med. Mal. Infect. 2016, 46, 177–187. https://doi.org/10.1016/j.medmal.2016.02.009.

56. Weil-Olivier, C.; Taha, M.K.; Emery, C.; Bouee, S.; Beck, E.; Aris, E.; Loncle-Provot, V.; Nachbaur, G.; Pribil, C. Healthcare Resource Consumption and Cost of Invasive Meningococcal Disease in France: A Study of the National Health Insurance Database. Infect. Dis. Ther. 2021, 10, 1607–1623. https://doi.org/10.1007/s40121-021-00468-w.

57. Taha, M.K.; Gaudelus, J.; Deghmane, A.E.; Caron, F. Recent changes of invasive meningococcal disease in France: Arguments to revise the vaccination strategy in view of those of other countries. Hum. Vaccin. Immunother. 2020, 16, 2518–2523. https://doi.org/10.1080/21645515.2020.1729030.

72. Antignac A, Ducos-Galand M, Guiyoule A, Pires R, Alonso JM, Taha MK. Neisseria meningitidis strains isolated from invasive infections in France (1999-2002): phenotypes and antibiotic susceptibility patterns. Clin Infect Dis. 2003, 37, 912-20. doi:10.1086/377739

73. Aubert L, Taha M, Boo N, Le Strat Y, Deghmane AE, Sanna A, et al. Serogroup C invasive meningococcal disease among men who have sex with men and in gay-oriented social venues in the Paris region: July 2013 to December 2014. Euro Surveill. 2015, 20. doi:10.2807/1560-7917.es2015.20.3.21016

74. Barret AS, Clinard F, Taha MK, Girard I, Hong E, Tessier S, et al. Cluster of serogroup W invasive meningococcal disease in a university campus. Med Mal Infect. 2020, 50, 335-41. doi:10.1016/j.medmal.2019.10.003

75. Cohen R, de La Rocque F, Aujard Y, Bingen E. [National surveillance of bacterial meningitis in children]. Arch Pediatr. 2003, 10 Suppl 1:114s-5s. doi:10.1016/s0929-693x(03)90405-2

76. Garnier F, Courouble M, Denis F, Ploy MC. Emergence of 2 Neisseria meningitidis serogroup C clones in a French county. Diagn Microbiol Infect Dis. 2011, 69, 280-2. doi:10.1016/j.diagmicrobio.2010.09.024

77. Grodet C, Dequin PF, Watt S, Lanotte P, de Gialluly C, Taha MK, et al. Outbreak in France of Neisseria meningitidis B:15:P1.12 belonging to sequence type 1403. Clin Microbiol Infect. 2004, 10, 845-8. doi:10.1111/j.1469-0691.2004.00935.x

78. Hong E, Terrade A, Muzzi A, De Paola R, Boccadifuoco G, La Gaetana R, et al. Evolution of strain coverage by the multicomponent meningococcal serogroup B vaccine (4CMenB) in France. Hum Vaccin Immunother. 2021, 17, 5614-22. doi:10.1080/21645515.2021.2004055

79. Levy-Bruhl D, Perrocheau A, Mora M, Taha MK, Dromell-Chabrier S, Beytout J, et al. Vaccination campaign following an increase in incidence of serogroup C meningococcal diseases in the department of Puy-de-Dome (France). Euro Surveill. 2002, 7, 74-6. doi:10.2807/esm.07.05.00368-en

80. Sarlangue J, Levy C, Cohen R, Bingen E, Aujard Y. [Epidemiology of bacterial meningitis in children in France]. Arch Pediatr. 2006, 13, 569-71. doi:10.1016/j.arcped.2006.03.016
